# The Chemistry and Pharmacology of Citrus Limonoids

**DOI:** 10.3390/molecules21111530

**Published:** 2016-11-13

**Authors:** Roberta Gualdani, Maria Maddalena Cavalluzzi, Giovanni Lentini, Solomon Habtemariam

**Affiliations:** 1 Department of Chemistry ”U. Shiff”, University of Florence, Via della Lastruccia 3, Florence 50019, Italy; roberta.gualdani@unifi.it; 2 Department of Pharmacy-Drug Sciences, University of Studies of Bari Aldo Moro, Via E. Orabona n. 4, Bari 70126, Italy; mariamaddalena.cavalluzzi@uniba.it; 3 Pharmacognosy Research Laboratories & Herbal Analysis Services, University of Greenwich, Central Avenue, Charham-Maritime, Kent ME4 4TB, UK

**Keywords:** citrus limonoids, tetranortriterpenoids, lead compound, structure-activity relationships, ligand efficiency metrics, developability, antimicrobial, anticancer, antioxidant, antiinflammatory, insecticidal, antidiabetic

## Abstract

Citrus limonoids (CLs) are a group of highly oxygenated terpenoid secondary metabolites found mostly in the seeds, fruits and peel tissues of citrus fruits such as lemons, limes, oranges, pumellos, grapefruits, bergamots, and mandarins. Represented by limonin, the aglycones and glycosides of CLs have shown to display numerous pharmacological activities including anticancer, antimicrobial, antioxidant, antidiabetic and insecticidal among others. In this review, the chemistry and pharmacology of CLs are systematically scrutinised through the use of medicinal chemistry tools and structure-activity relationship approach. Synthetic derivatives and other structurally-related limonoids from other sources are include in the analysis. With the focus on literature in the past decade, the chemical classification of CLs, their physico-chemical properties as drugs, their biosynthesis and enzymatic modifications, possible ways of enhancing their biological activities through structural modifications, their ligand efficiency metrics and systematic graphical radar plot analysis to assess their developability as drugs are among those discussed in detail.

## 1. Citrus Limonoids: An Introduction

### 1.1. Complexity and Evolution of Citrus Limonoid Research

**Figure 1:**
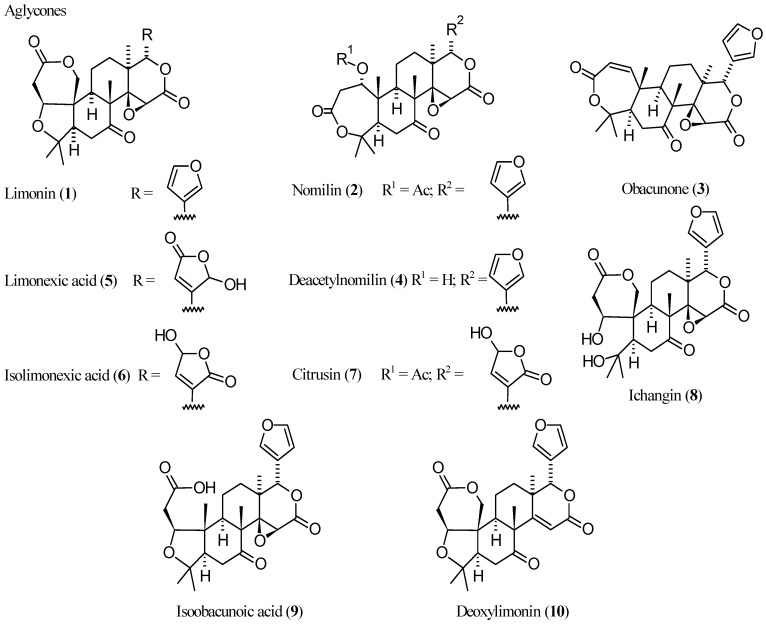

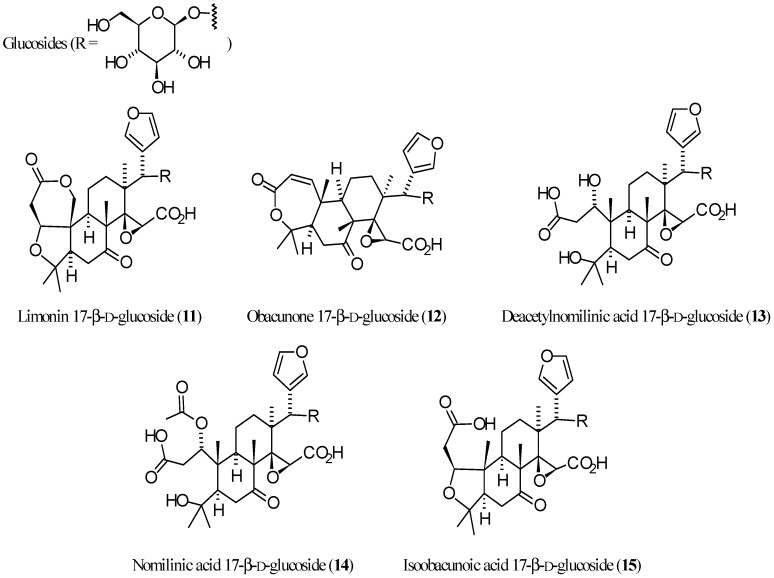
Structures of citrus limonoids (CLs) of the limonin (**1**) biosynthetic group (aglycones and corresponding β-d-glucosides) studied in the stated period (2005–2016).

Citrus limonoids (CLs) are a crowded family of polycyclic secondary metabolites found mostly in seeds [1], fruits [2], and peel [3,4,5,6,7] tissues of citrus fruits, including lemons, limes, oranges (both sour and sweet), pumellos, grapefruits, bergamots, and mandarins [5]. CLs are regarded as highly oxygenated triterprenoids as they contain relatively high numbers of oxygen atoms (7–11) in their structures. CLs are found as both free aglycones and corresponding β-d-glucosides, the former mostly occurring in seeds while the latter are formed during fruit maturation. The tissue distribution of aglycones and corresponding glucosides reflect their solubility in water with relatively more insoluble compounds, free CLs, being more abundant in seeds and fruit peel [5]. The formation of CL β-d-glucosides is catalyzed by a specific limonoid glucosyltransferase that reduces the concentration of bitter limonoid aglycones in citrus fruits thus conferring agreeable taste to citrus fruit juices and other products [1,8]. Indeed, several CLs gradually confer alkaloid-like taste to citrus fruit juices after processing (delayed bitterness [9]). With the notable exception of some ceremonial alcoholic beverages (social lubricants such as cocktails), the quinine-like bitter taste is generally not favoured by humans. Thus, early studies on CLs focused on enhancing the commercial value of fruit juices by employing debittering methods that reduce the content of these secondary metabolites [1]. In the last decade, delayed bitterness has continued to attract interest from several research groups and a number of papers have been dedicated to studies on mechanisms underlying delayed bitterness [10,11,12,13,14], possible ways of reducing [15,16,17,18] or modulating [19,20,21,22,23] the formation of bitter limonoids [1] in citrus fruits and juice products. On the other hand, evaluation of biological activities of CLs have disclosed great potential for these phytochemicals that furnished the rational basis of traditional medicinal uses of CL-containing folk remedies [24,25,26,27,28,29] as well as modern nutraceutical products [29,30]. Obviously, taste is a biological answer to xenobiotics just like any other pharmacological effect and during studies on delayed bitterness, a new limonoid possibly endowed with peculiar biological activities was discovered (see Section 3). Thus, considering the two sets of studies (bitterness vs. other pharmacological effect) as being separated is nothing more than an *escamotage* suggested by the sake of simplicity and the need to reduce the dimensionality of treatise. Indeed, this review will not consider details of recent literature concerning the problem of delayed bitterness and its negative economic impact on the citrus industry and hence the reader may refer to the above cited references. In the following sections, we systematically review the limonoids of the limonin (**1**, Figure 1) group (and corresponding β-d-glucosides) [8] which were studied in the last decade (2005–2016), focusing on analytical and medicinal chemistry aspects. Newly isolated limonoids will be reported and a wide (but not exhaustive) overview on recent findings concerning limonoid bioactivity will be given. Further references focusing on limonoid biological activities may be found in several excellent previous reviews [1,15,29,30,31,32,33,34,35,36]. Most of the references reviewed herein derive from Reaxys database. Further references were obtained through browsing PubMed, ScienceDirect and Google by substance chemical names. Patent literature was not considered.

### 1.2. Chemistry and Classifications

The chemistry of CLs both in a historical perspective [29] and for structural classification purposes [37] has been masterfully treated in recent reviews. Briefly, CLs are classified in the enlarged class of the terpene family of natural products. Terpenes (from terpentin, old spelling of turpentine, C_10_H_16_ [38]) are natural products formally deriving from head-tail polymerization of isoprene (C_5_H_8_) units [39]. The two common isoprene building blocks: isopentenyl pyrophosphate (IPP) and dimethylallyl pyrophosphate (DMAPP) enzymatically-polymerise to form triterpene precursors like that of squalene. Thus, six isoprene units are needed to form the triterpene squalene (C_30_H_50_), the closest biosynthetically CL-related hydrocarbon. The processing of such a triterpene skeleton through reactions including cyclization, aromatisation, rearrangements, and oxygenation can give rise to around 200 different sub-structural groups, including the highly oxygenated subgroup of CLs. Since an isobutyl terminal moiety is missing in all CLs, they are generally named tetranortriterpenoids, where the prefix “tetranor-“ indicates the loss of four carbon atoms [40].

**Figure 2:**
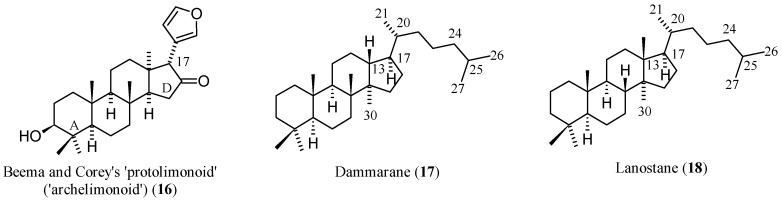
Structures of reference compounds for citrus limonoids (CLs).

Nature gave a formidable representation of her fantasy when developing limonoid structures. The variety and complexity of scaffolds found in the limonoid family constitute the foundation for the wide range of biological activities shown by these compounds and represent a challenge for researchers who want to study structure-activity relationships (SAR) or, more modestly, delineate relationships based on shared structural features. In the latter case, it is a common practise to relate the structure of limonoids to an archetypal limonoid scaffold from which actual limonoids may be formally derived modifying cycles and/or introducing or modifying functional groups and substituents. This reference compound (**16**, Figure 2) consists of four rings condensed in steroid-like manner and designated A through D [37]. At the *C*-17 position, a furan ring is attached while five methyls are positioned at *C*-4, two β- and one α-hydrogens at *C*-8, *C*-10, and *C*-13 respectively. The compounds 13-*epi*-24,25,26,27,30-pentanordammarane and 13-*epi*-24,25,26,27,30-pentanorlanostane may be considered compound **16**’s closest parent hydrides (cf. the dammarane (**17**) and lanostane (**18**) structures in Figure 2).

The term “protolimonoid” [37,41] to indicate reference molecular entities like **16** should be discouraged since properly called protolimonoids (also named protomeliacins or melianes [42]) are (a) naturally occurring compounds; (b) bear an intact (eventually oxygenated) dammarane-like *C*-17 side chain [43]; and (c) seem to be biogenetically related to limonoids as biochemical precursors [42,43,44], while the synthetic compound **16** [41] that bears a furan ring at position *C*-17 has not been detected in the plant kingdom.

During the biosynthetic processes, all CLs of the limonin group (Figure 1) have undergone oxidative fission at ring A. On this basis, they are defined as A-secolimonoids, with the prefix “seco-“ (from Latin *secare*, to cut) denoting ring cleavage. Several of them have also undergone bond cleavage at the ring D. Counterintuitively, CLs present more rings than compound **16**. This happens for two reasons: (1) in all CLs but **10**, an oxirene ring is fused on the D ring; (2) oxidative cleavage of *C*-4,*C*-5- and *C*-16,*C*-17-bonds, and eventual further oxidation generate carboxyl and hydroxyl groups that are condensed to generate lactones rings. This is why CLs are generally referred to as mono- or dilactones. In congeners **5**–**7**, the furan ring appended to the *C*-17 position is further oxidised to give hydroxybutenolide moieties which confer mild acidic properties. Table 1 offers an overview of the main structural features of the stated compounds.

Where free carboxylic groups are present, higher acidic properties arise. This is the case of isoobacunoic acid (**9**) and all β-d-glucosides which are formally obtained in a two-step route including ring D lactone opening and subsequent condensation with β-d-glucose. Thus, compounds **1**–**15** may be classified in increasing order of acidity, from neutral (**1**–**4**, **8**, **10**) through the most acidic congeners (dicarboxylic acid β-d-glucosides). Table 2 reports p*K*_a_ value ranges covering the acidity of compounds **1**–**15** together with other main physico-chemical features for the stated compounds. Most studied biological activities (discussed in detail in Section 3) are given as well. As expected, aglycones present the highest log *P* values while glucosides show negative log *D*_7.4_ values thus indicating high hydrophilicity at the physiological pH. The unbalanced solubility profile of the latter may explain the relatively low number of studies on their respective biological activities which are hampered by difficult isolation from natural sources and poor pharmacokinetics.

### 1.3. Biosynthesis and Enzymatic Modifications

**Figure 3:**
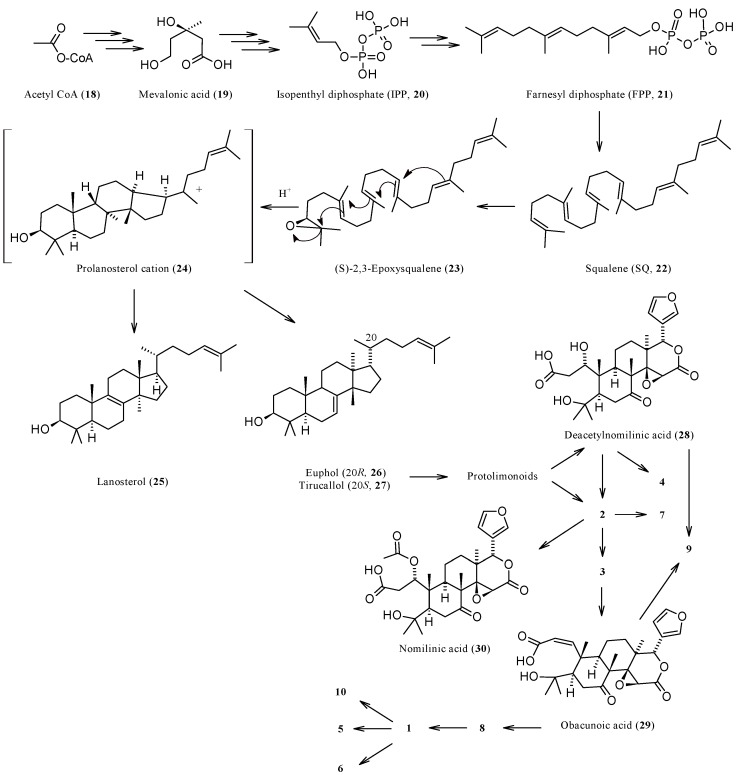
Precursors of citrus limonoids and proposed biogenetic relationships between congeners of the limonin (**1**) group.

The biochemistry of CLs has been adequately reviewed in the past [8,15,90] and will not herein be considered in detail. A graphical overview of the main biosynthetic routes involving the CLs is presented in Figure 3. The farthest CL precursor is obviously acetyl coenzyme A (**18**) which gives mevalonic acid (**19**) through a three-step biosynthetic route. Three further steps are needed to obtain isopentyl diphosphate (IPP, **20**) or its isomer dimethylallyl diphosphate (DMAPP). Three C5 units of IPP/DMAPP furnish carbons to constitute the scaffold of farnesyl diphosphate (FPP, **21**) and two C15 units of the latter finally give squalene (SQ, **22**), the closest hydride to CLs. SQ is oxidized to give a reactive epoxide, (*S*)-2,3-oxidosqualene (also named 2,3-epoxysqualene or squalene epoxide, **23**), which undergoes cyclization generating a prolanosterol cation **24**. Through a series of 1,2-shifts of hydrides and methyls, this intermediate may generate several triterpenes including lanosterol (**25**) and the two epimers euphol (**26**) and tirucallol (**27**). The latter lanostadienols are both considered as the ultimate biogenetic precursors of CLs. Indeed, dramatic oxidation of the *C*-17 side chain of **26** and **27** generate several protolimonoids which finally give nomilin (**2**) and deacetylnomilinic acid (**28**), the two CLs likely precursors of all limonoids of the limonin group. The proposed biogenetic relationships between compounds **1**–**15** are graphically summarized in Figure 3 and briefly described in the next section. The reader may also find further details on the biosynthesis of these secondary metabolites in excellent recent reviews [37,43].

## 2. Limonin Congeners: A Medicinal Chemistry Perspective

### 2.1. Medicinal Chemistry Tools

One must appreciate that a simple overview of biological activities documented for CLs over the last decade would not be sufficient to scrutinise the compounds’ therapeutic and/or lead potential. Hence, a comprehensive medicinal chemistry tools and analysis methodology are employed herein to assess the various limonin biosynthetic products. Degraded limonoids (e.g., isobenzofuranones sharing structural features with limonoids [91]) and other tetranortriterpenoids extracted from different genera and/or not belonging to the limonin biosynthetic group will not be considered and hence readers are rather directed to relevant recent reviews [15,37,92,93,94,95,96,97,98,99,100,101]. 

From the medicinal chemistry point of view and to envisage best CLs candidates as lead compounds, ligand efficiency metrics (LEM) need to be calculated. LEM [102] are composite parameters [103] ever increasingly used to set expectations about lead compound developability [104]. The most relevant LEM indices adopted herein along with their corresponding brief explanations [105] are summarised below:

- Lean ring index (LRI) = heterocycles/carbocycles ratio. This index was introduced since the number of aromatic and heteroaromatic rings has been found to correlate with an increased risk of attrition in development [106]. In particular, the following scale in the detrimental effects of rings has been observed [107]: carboaromatics >> heteroaromatics > carboaliphatics > heteroaliphatics, with the latter generally being beneficial. Thus, the higher LRI, the better the developability.
- Potency efficiency index percentage (PEI%) = (potency/MW Da) × 100, where potency is generally given in the form of –Log (half maximal inhibitory concentration) (pIC_50_) or –Log (half maximal effective concentration) (pEC_50_) for the activity actually concerned. We adopted this index as a slight modification of the well-known binding efficiency index (BEI) = pIC_50_/MW kDa [104] since PEI% values allow clearer graphical representations than the former. Since an idealized compound should have a BEI value of 27, the corresponding PEI% value would be 2.7.
- Ligand efficiency index (LEI) = potency/number of non-hydrogen atoms (“heavy atoms”, HA). For the sake of graphical clarity, we adopted the descriptor LE (ligand efficiency) corresponding to the product LEI × 1.37 (LE = ∆G°/HA = −2.303 RT/HA ≈ 1.37 potency/HA). An ideal lead compound should have LE > ~0.3 kcal per mole per HA [102].
  Fraction sp^3^ (Fsp^3^ = number of sp^3^ hybridized carbons/total carbon count). Lovering [108] suggested this descriptor as a measure of complexity since he noted that drug candidates nearest to clinical use generally present high Fsp^3^ and chirality centers. 0.36 is the suggested lowest allowed limit value [109].
- Lipophilic ligand efficiency (LLE = potency − cLog *P*, also referred to as lipophilic efficiency, LipE). LLE is simply the difference between potency and the most popular lipophilicity descriptor (cLog *P*), and may be considered as a measure of the specificity of binding. Proposed acceptable values of LLE for drug candidates are >~5 [102].
- Ligand efficiency dependent lipophilicity (LELP = cLog *P*/LE). LELP combines aspects of LE and LLE into a single index and may be considered as the price of ligand efficacy paid for with Log *P* [110]; it should be <7.5 [102].

By using these analysis parameters, recent attempts undertaken to enhance the biological activities of natural limonoids through structural modification (i.e., semisynthetic analogue preparation and bio-evaluation) are analysed in the forthcoming text.

### 2.2. Citrus Limonoids: Sources, Main Structural Features, and Developability As Therapeutic Agents

#### 2.2.1. Limonin (**1**, Figure 1)

Known in the past also as obaculactone, evodin, or dictamnolactone from the names of some of its natural sources (*Phellodendron amurense* bark—Phellodendri Cortex, Ōbacu in Japanese [111]—*Dictamnus albus* extracts, and *Evodia rutaecarpa* fruit, respectively) [112], limonin (**1**) is universally considered as the standard bearer of CLs and continues to be the most studied congener of the class. Limonin (**1**) is normally extracted from the *Citrus* genus plant fruits and hence it’s early trivial name, citrolimonin [112]. “Limonin”, in turn, recalls limon*—*the transliterated form of the old English word *lymon* which came from old French *limon* (“citrus fruit”; via Provençal, from Persian *limu* passing through Arabian *laimun*, generic terms for citrus fruits, cognate with Sanskrit *nimbu*) [113]. When **1** was discovered [114], its bitter taste and some preliminary assays disguised it as an alkaloid probably suggesting to Berlays [115] the stem—*in(e)* which was commonly used for quinine-like compounds. Limonin has also recently been found in *Citrus* genus plant bark [25,116,117], and root [118], tissues. In addition to the above reported plants, **1** has been isolated from numerous non-*Citrus* species such as the endemic Brazilian species *Raulinoa echinata* [119], *Glycosmis parva* (a wild small shrub distributed in Thailand) [120], *Poncirus trifoliata* (a tree that is widely distributed in China) [121], and *Dictamnus angustifolius* [27,122,123].

**Figure 4:**
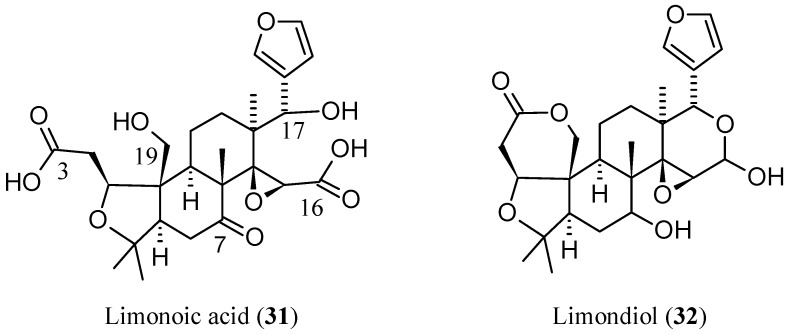
Structures of limonoic acid (**31**) and limondiol (**32**).

From a biosynthetic point of view (Figure 3), **1** has nomilin (**2**) as its most accredited parent compound in an enzymatic route possibly passing through obacunone (**3**), obacunoic acid (**29**), and ichangin (**8**). Despite extensive radioactive tracer work, the definitive proof on all four involved steps has not yet been given. Alternatively, isoobacunoic acid (**9**) was once considered as a possible precursor of **1**. Unfortunately, radioactive **9** has not been shown to be converted to **1** in *Citrus* [8]*.* Structurally, **1** may be considered as the dilactone derivative of limonoic acid (**31**, Figure 4) and hence can be given semisystematic names such as limonoic acid di-δ-lactone, limonoic acid 3,19:16,17-dilactone, or limonoate A-ring, D-ring dilactone. On the other hand, **1** also relates to the oxidation product, limondiol (**32**, Figure 4), and thus may also be named as 7,16-dioxo-7,16-dideoxylimondiol.

When dissecting the fused hexacyclic scaffold of **1**, the isochromene (2-benzopyran) system is the higher priority parent component, followed by isobenzofurane, pyran, and oxyrene rings, in decreasing order of priority. In comparison with the archetypal limonoid scaffold 4,4,8-trimethyl-17-(furan-3-yl)androstane (**16**, Figure 2), two out of four fused carbocycles (cycles A and D) are replaced by a furopyran and a pyran system, respectively, which are condensed to a surviving naphthalene core.

Due to its good number of chiral centres (nine) and its high fraction sp^3^ (Fsp^3^ = 0.73), **1** is one of the most complex CLs. The unusually high number of heterocycles (LRI = 2.5) together with its relatively low lipophilicity (cLog *P* = 1.66) render the ligand metrics profile of **1** relatively good. This consideration together with the relatively high availability of **1** in adequate amount to facilitate the preparation of semisynthetic analogues endorse **1** as a lead compound and justify both the plethora of biological activities reported for **1** (cf. Table 2 and Section 3) and its universally recognised role of flagship member of the natural polyoxygenated triterpene family [37].

**Figure 5:**
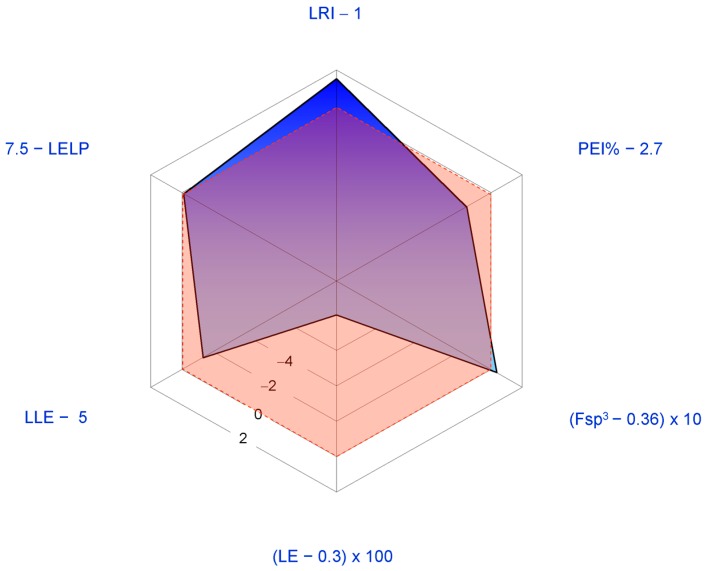
Radar plot of ligand efficiency metrics as a graphical tool to assess developability of limonin (**1**) as a drug. Sub-optimal property space corresponds to the inner red hexagon showing sides marked with “zero”. Ideally, good lead compounds would be represented by areas wider than this inner hexagon. This spider plot indicates the properties of **1** that need improvement. Legend: Fsp^3^: fraction sp^3^; LRI: heterocycles/carbocycles ratio; PEI: potency efficiency index; LE: ligand efficiency; LLE: lipophilic ligand efficiency; LELP: ligand efficiency dependent lipophilicity; see text for details.

The most studied pharmacological activities of **1** are related to its possible application as an anticancer agent. Generally **1** displays antiproliferative activity in the low micromolar range. Thus, the structure of **1** is relatively redundant when considered in relation to its displayed potencies (LE < 0.37 Kcal per mol per HA; PEI% < 2.7). In Figure 5 a spider plot based on LEM calculated for **1** is shown where the IC_50_ value for in vitro aromatase inhibition (5.22 μM [46]) was used as a measure of potency.

**Figure 6:**
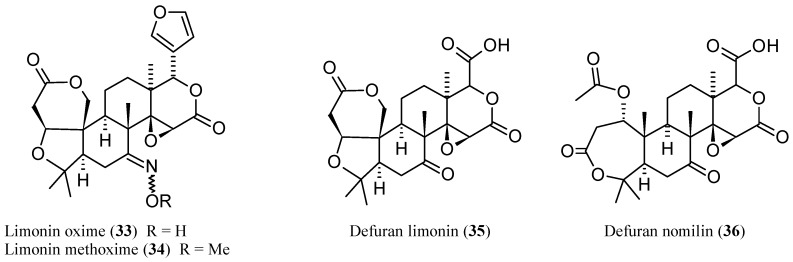
Semisynthetic analogs of limonin (**1**); and nomilin (**2**) endowed with anti-aromatase (**33**–**36**) [46]; and anti-biofilm (**33**–**35**) [63] properties.

The radar plot indicates that **1** may be considered as a fairly good lead compound with the tactics of molecular simplification (i.e., reduction of molecular weight and HA) and introduction of nitrogen atoms (possibly improving potency) being obviously suggested. Both tactics have been recently explored with the former having inspired the synthesis of an ever increasing number of degraded limonoids [37,91]: a detailed examination of these compounds is however beyond the scope of this review. The latter tactic has been adopted in a relatively smaller number of studies. The straightest way to introduce nitrogen atoms in the limonin structure is represented by oxime derivatization of its *C*-7 carbonyl group. Patil and co-workers obtained limonin oxime (compound **33**, Figure 6) and its *O*-methyl derivative **34** [46]. Both compounds were about twice as potent as **1** in the aromatase inhibition assay. Incidentally, defuranlimonin (**35**, a limonin analogue with a carboxyl group appended at the *C*-17 position in lieu of the furan ring; Figure 6) was as active as **1** while defuran nomilin (**36**) was about six times more potent than **2**. A similar trend was observed when studying the effect of CLs on biofilm formation in *E. coli* where **33**, **34**, and **35** were more active than **1** [63]. Furthermore, **34** showed higher induction of the detoxicant liver enzyme glutathione S-transferase (GST) than the one displayed by **1** [49]*.* These findings suggest the possibility of improving potency by introducing nitrogen atoms in the CL scaffold and underlay the structural redundancy of CLs. However, pitfalls in this generalization should always be taken into account. When inhibition of cell-cell signalling was investigated in *Vibrio harvey*, **1** resulted to be more active than its derivatives **33**–**35** [63]. The variability of the in vitro study results is one of the challenging aspects in the study of CL activities and was also noted when studying proapoptotic [89] and cytotoxic effects [81] of CLs on eukaryotic cells. Puzzling as it is, the finding of varying effects of CLs on different cell lines suggests the possibility that mixtures of CLs, such as those found in citrus fruits, may prove more effective with respect to the isolated components when trying to face highly varying human cancers [81] or polybacterial human diseases.

**Figure 7:**
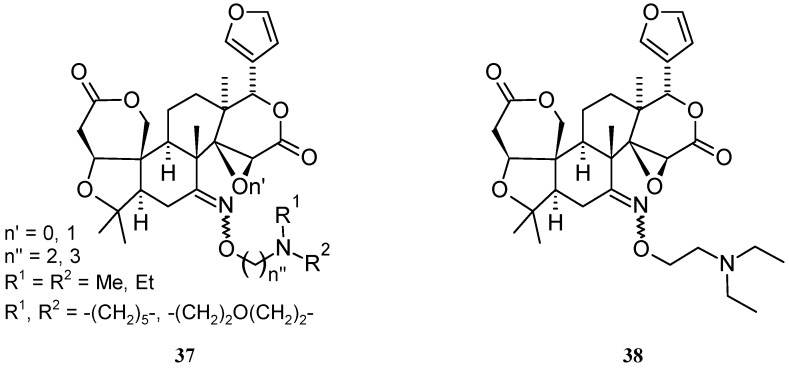
Water-soluble analogs of limonin (**1**); and deoxylimonin (**10**) endowed with analgesic and anti-inflammatory properties [45].

A series of limonin (**1**) and deoxylimonin (**10**) oxime derivatives of the general structure **37** (Figure 7) were obtained through an oxime derivatization procedure, and subsequently evaluated as anti-inflammatory (ear swelling induced by xylene test in mice) and analgesic (acetic acid-induced writhing and tail-immersion tests in mice) agents [45]. In this case, tertiary amine moieties were introduced onto *C*-7 position in order to obtain water-soluble derivatives, possibly endowed with higher bioavailabilities than those displayed by the parent CLs **1** and **10**. The latter were chosen as parent compounds to evaluate the role played by the epoxide ring in the above activities. Generally, **1** and its derivatives were more active than the corresponding deoxygenated congeners, thus underlying the relevant contribution of the oxirene moiety to analgesic and anti-inflammatory activities. Whether this contribution stems from epoxyde reactivity towards protein nucleophilic side chains [3] however remains to be demonstrated. Incidentally, these findings are in agreement with the above general observation on LRI, Fsp^3^, and the number of chirality centers as reliable predictors of drug developability. As expected, all derivatives were more water-soluble than the parent compounds. Water-solubility was obviously directly related to p*K*_a_ values and inversely related to Log *D*_7.4_. The most interesting compound was the limonin derivative bearing an *O*-diethylaminoethyl side chain (**38**) since it was more potent than acetylsalicylic acid and naproxen in the analgesic and the anti-inflammatory assays, respectively.

Generally, **1** and its glucoside **11** share the same activities regardless of the test used (cf. Table 2). However, relevant nuances in favour of **11** have been reported. As an example, both the aglycon and its glucoside are more effective against neuroblastoma cells (SH-SY5Y) compared to colon carcinoma cells (Caco-2) while having no effect on normal cells (epithelial Chinese hamster ovary cells, CHO) [58]. However, **1** showed a slower rate of induction of caspase 3/7 activity in comparison to **11**. Furthermore, while micromolar concentrations of both **1** and **11** arrested cell growth, biochemical and morphological data showed that **11** induced a more rapid cell death. Incidentally, the higher resistance of Caco-2 cells may be related to high expression of the ABC transporter P-gp [3,51], an efflux pump that impedes cytoplasmic accumulation of lipophilic compounds.

#### 2.2.2. Nomilin (**2**, Figure 1)

Nomilin (**2**) is characterized by a seven-membered oxepine ring and may be considered as the product of an acetate equivalent addition to the structure of obacunone (**3**). The trivial name of **2** is an anagram of “limonin” while its semisystematic name is 1-acetyloxy-1,2-dihydroobacunoic acid ε-lactone, and is based on the trivial name for the product formally obtainable by hydrolysis of **3**, obacunoic acid (**29**). However, **2** precedes **3** and **29** in the proposed biogenetic relationships between CLs (Figure 3). Indeed, it has been hypothesized that trans-elimination of the acetyl group of **2** gives **3**, and the hydrolytic opening of the Α ring lactone of **3** gives **29**. The enzymes responsible for these two steps have been isolated from bacterial cells, but they have not yet been found in *Citrus* species [8]. The direct precursor of **2** is known to be deacetylnomilinic acid **28**.

**Figure 8:**
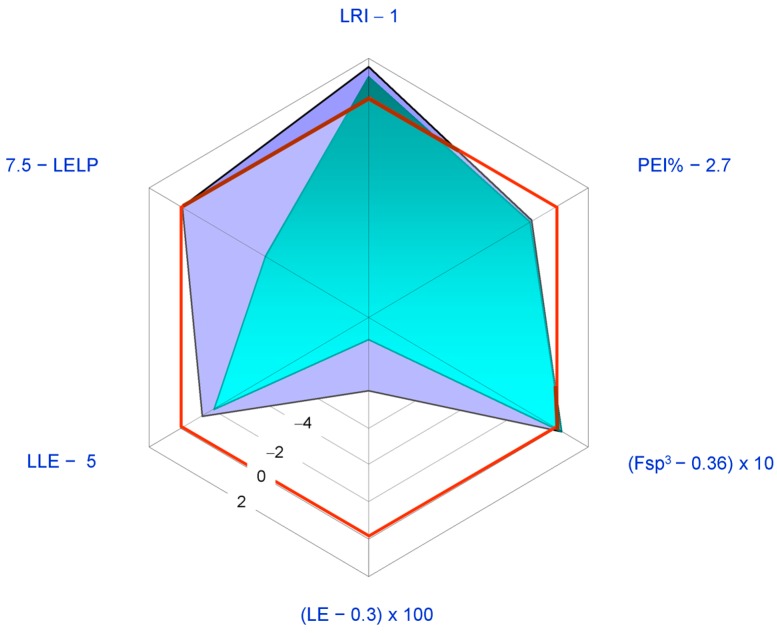
Radar plot of ligand efficiency metrics as a graphical tool to assess developability of limonin (**1**, purple area) and nomilin (**2**, blue-green area) as drugs. Sub-optimal property space corresponds to the inner hexagon showing red sides marked with “zero”. Ideally, good lead compounds would be represented by areas wider than this inner hexagon. This spider plot indicates that **1** should be preferred to **2** as a lead compound. Legend: Fsp^3^: fraction sp^3^; LRI: heterocycles/carbocycles ratio; PEI: potency efficiency index; LE: ligand efficiency; LLE: lipophilic ligand efficiency; LELP: ligand efficiency dependent lipophilicity; see text for details.

Radioactive tracer work on CLs is indebted to **2** since radiolabelled nomilin can be easily prepared using labelled acetate and in turn converted into other labelled CLs [8]. Compounds **1**–**4** are the major CLs and **2** has also recently been found in the barks of *Citrus* genus [7,25] and in root bark of *Dictamnus angustifolius* [122,123]. When biological activities are considered, **2** shares the same profile of **1** (Table 2) displaying comparable potencies in a range from five-fold lower to five-fold higher values than the ones shown by **1** [46,67,77]. Since **2** has higher MW, HA, and cLog *P* values than **1**, the latter seems to be preferable as a lead compound (Figure 8). For these comparative analysis, IC_50_ value from in vitro aromatase inhibition (18.86 μM [46]) was used as a measure of potency.

#### 2.2.3. Obacunone (**3**, Figure 1)

This α,β-unsaturated ε-lactone is also known as obacunon (cf. **1** for ethymology), casimirolide (from *Casimiroa edulis*, Mexican apple) or tricoccin S3 (since initially believed to be cognate of tricoccins—secondary metabolites isolated from *Cneoraceae* species such as *C. tricoccon*). Compound **3** may be obtained by refluxing **2** with a mixture of acetic anhydride and pyridine [124] and may be considered as the product of intramolecular condensation of obacunoic acid **29** (obacunoic acid, ε-lactone; limonoic acid 3,19:16,17-dilactone, limonoate D-ring-lactone). Indeed, **29** was obtained by heating **3** in a 0.1 M NaOH solution [124].

Radioactive tracer work demonstrated the biosynthetic route relating **3** to **2**, as a product, and to **29**, as a precursor [8] (Figure 3). In the last decade, **3** has been isolated from non-*Citrus* species, including *Phellodendron amurense* [125], *Dictamnus dasycarpus* [27,126,127], *Dictamnus angustifolius* [27,122,123], and *Harrisonia perforata* (the only species of the genus *Harrisonia* growing in Thailand) [128].

**Figure 9:**
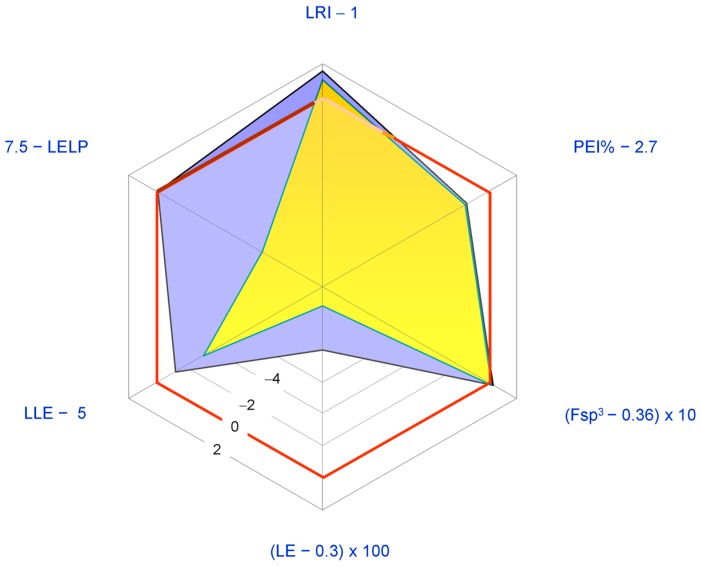
Radar plot of ligand efficiency metrics (LEM) as a graphical tool to assess developability of limonin (**1**, blue area) and obacunone (**3**, yellow area) as anticancer agents. Sub-optimal property space corresponds to the inner hexagon showing red sides marked with “zero”. Ideally, good lead compounds would be represented by areas wider than this inner hexagon. This spider plot indicates that **1** should be preferred to **3** as a lead compound. Legend: Fsp^3^: fraction sp^3^; LRI: heterocycles/carbocycles ratio; PEI: potency efficiency index; LE: ligand efficiency; LLE: lipophilic ligand efficiency; LELP: ligand efficiency dependent lipophilicity; see text for details.

The anticancer activity of **3** has been mostly attributed to the α,β-unsaturated ketone functional group in the A ring [15]. The high lipophilicity of **3** (cLog *P* = 2.91, Table 2) should also be taken into account. In fact, this property is generally positively related to cytotoxicity since the kinetics of drug uptake in cancer cells is higher with increased lipophilicity [129]. However, **3** rarely came so far as the most active potential anticancer CL of the limonin group [46] in the stated period, when anticancer activity is concerned. Thus, the price paid in term of Log *P* to gain activity through lipophilicity was not a good investment with **3** and its LEM profile acknowledges it being inferior to **1** as a lead compound for anticancer design (Figure 9). For this analysis, the IC_50_ value for in vitro aromatase inhibition (28.04 μM [46]) was used as a measure of potency.

**Figure 10:**
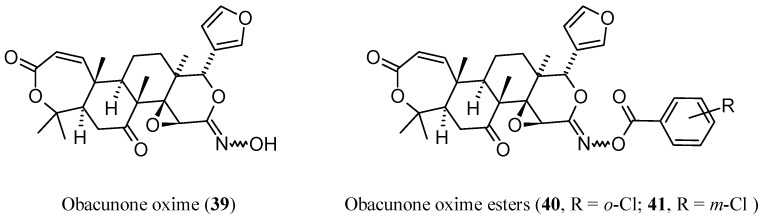
Structures of obacunone oxime (**39**) and two of its ester derivatives (**40**,**41**) endowed with high larvicidal activity [80].

The tactics of nitrogen introduction via oxime derivatization of **3** has recently led to a series of obacunone oxime and its ester derivatives were obtained (Figure 10) [80]. Furthermore, the larvicidal activity of these compounds against oriental armyworm (*Mythimna separata* Walker), a lepidopteran pest, has been evaluated [80]. Rewardingly, several of the studied compounds were more active than the lead **3**, with the two chlorobenzoyl derivatives **40** and **41** displaying 60% higher larvicidal activity than that of the parent compound **3**.

#### 2.2.4. Deacetylnomilin (**4**)

At the time of its early isolation from orange seeds, deacetylnomilin (**4**) was erroneously considered as an isomer of limonin (**1**) and called isolimonin [130,131]. Indeed, **4** may be considered as the formal product of reductive cleavage of the A ring lactone of **1** and subsequent isomerization of the so-obtained carboxylic acid (isoobacunoic acid, **9**) to give a β-hydroxy-ε-lactone ring (4-hydroxyoxepin-2-one) in lieu of the tetrahydrofuran one. Thus, two hydrogen atoms should be added to **1** structure in order to obtain **4** (see Table 1). The relationship between **4** and **2** is reflected in their corresponding semisystematic names since **4** is also known as 1,2-dihydro-1-hydroxyobacunoic acid, ε-lactone. A straighter structural linkage relates **4** to deacetylnomilinic acid **28**, which is most likely the initial precursor of all limonin related CLs discussed in this review [8] (see Figure 3).

The physico-chemical profile of **4** shows a definite overlapping with that of **1**. No surprise, then, if the two CLs present similar activities and potencies of action [3,47,51,58,77]. Paralleling what has been observed for **1** and its glucoside **11**, the glucoside of **4** (**12**) sharply reduced cancer cell viability as compared to aglycone [58] thus corroborating the hypothesis that glucosides may be better active apoptosis-inducing agents [132].

#### 2.2.5. Pseudoacids: Limonexic Acid (**5**), Isolimonexic Acid (**6**), and Citrusin (**7**)

Limonexic acid (**5**) may be considered as a product of oxidation of the furan ring of **1** to give a Υ-hydroxybutenolide moiety appended at *C*-17. Since limonoids with oxidized furan rings are relatively rare in the *Rutaceae*, this 21-hydroxy, 23-oxo analogue of **1** has long been considered as an artefact caused by photooxidation occurring during extraction of **1** from its natural sources. Indeed, photooxidative degradation of the furane ring may cause limonin glucoside instability in vitamin B_2_-containing beverages exposed to light [133] and oxidative cleavage is a commonly used approach to obtain degraded limonoids [92]. When the constitutional isomer of **5**, isolimonexic acid **6**, was isolated from *Tetradium glabrifolium* [134], the possibility that **5** and **6** were real secondary metabolites was considered as probable since spontaneous photooxidation reactions may hardly generate stereochemically homogeneous, isolated isomers such as the above butenolides [119]. Unfortunately, an error occurred in the graphics of the first work reporting on **6** [134] and this generated some confusion in the literature to the point that the Chemical Abstracts Service (CAS) gave two different CAS numbers to the above isomers but these numbers correspond to the same structure while PubChem attributes the same SMILES notation to **5** and **6**. In 2001, Gai et al. reported on the isolation of “a new compound” from *Evodia rutaecarpa*, 21-(*R* and *S*)-hydroxy-23-oxo-20-en-limonin, and named it shihulimonin A (Shihu is a word frequently found in the Chinese folk medicine but is commonly related to *Dendrobium* genus) [135]. Indeed **5** was early isolated by Emerson and generally referred to as ‘Emerson’s substance X’ [131]. The agreeing works of several research groups [136,137,138] definitively demonstrated that **5** has the structure reported in Figure 1 while **6** is the 23-hydroxy, 21-oxo isomer of **5**, notwithstanding contrasting literature graphics [25,28,118,120]. The hemiacetal C-atom (*C*-21 and *C*-23 for **5** and **6**, respectively) is labile in solution and this is why no stereochemically notations are generally reported about them. Incidentally, the stem -exic is used in lieu of -oic to indicate butenolide pseudoacids. Probably this derives from the fact that “exic” in Japanese stays for “eic”, thus indicating the α,β-unsaturated nature of the pseudoacid ring.

Limonexic acid (**5**) has been recently found also in *Citrus* genus plant barks [25,117], root [118], and flower [28] tissues. Other natural sources of **5** in the stated period were *Raulinoa echinata* Cowan (a spiny shrub endemic of Brazil) [139,140] and *Glycosmis parva* (a wild small shrub distributed in Thailand; a mixture of **5** and **6** was obtained) [120]. Isolimonexic acid (**6**) has been isolated also from *Citrus* genus plant root [118].

A relatively less explored pseudoacid is citrusin (**7**). Biogenetically derived from nomilin (**2**), this Υ-hydroxybutenolide is to **2** as isolimonexic acid (**6**) is to **1**. Citrusin has been recently found also in *Citrus* genus plant bark [25]. The biosynthetic relationship between the pseudoacids **5**–**7** and the corresponding putative parent compounds **1** and **2** is to be demonstrated.

**Figure 11:**
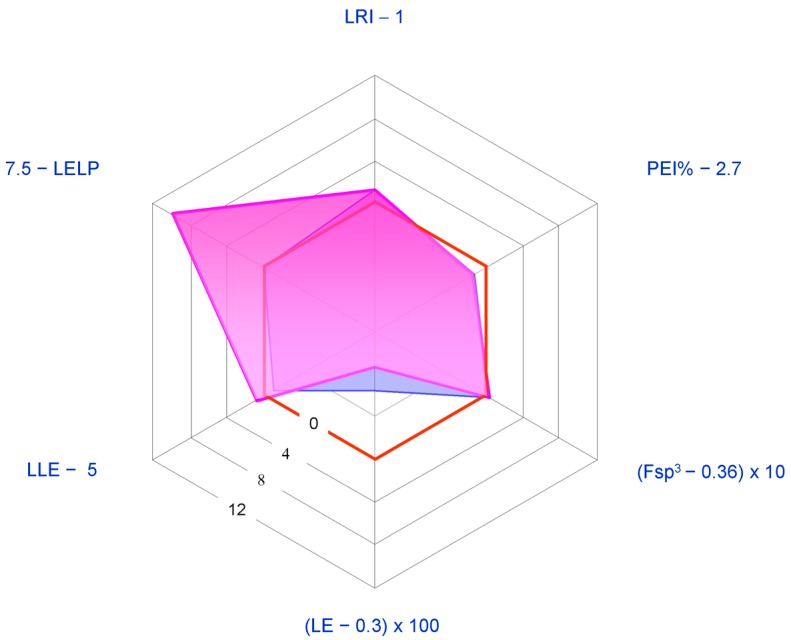
Radar plot of ligand efficiency metrics (LEM) as a graphical tool to assess developability of limonin (**1**, blue area) and limonexic acid (**5**, magenta area) as anticancer agents. Sub-optimal property space corresponds to the inner hexagon showing red sides marked with “zero”. Ideally, good lead compounds would be represented by areas wider than this inner hexagon. This spider plot indicates that **5** should be preferred to **1** as a lead compound. Legend: Fsp^3^: fraction sp^3^; LRI: heterocycles/carbocycles ratio; PEI: potency efficiency index; LE: ligand efficiency; LLE: lipophilic ligand efficiency; LELP: ligand efficiency dependent lipophilicity; see text for details.

Considering the presence of one more chiral centre, the absence of aromatic ring, and low lipophilicity (cLog *P* < 0), pseudoacids **5**–**7** are probably the most interesting CLs as lead compounds for further semisynthetic analogue development. In particular, an introduction of an aromatic ring would be well tolerated and should in principle allow increased activity. In Figure 11, the LEM profile of **5** is reported in comparison with that of **1**. The IC_50_ value for in vitro aromatase inhibition (20.02 μM [46]) was used as a measure of potency. The studies conducted so far suggest that **5**–**7** are not superior to **1** or **2** (cf. Section 3).

Ichangin (**8**) (from *C. ichangensis*) has also been recently isolated from the stem bark and leaves of the South-Americaan plant, *Raputia heptaphylla* [141]. It may be considered as the product of hydrolytic cleavage of the oxygen-containing portion of the isobenzofuran nucleus of **1** (A’ ring). The removal of A’ ring creates a spiro junction at the *C*-10. Indeed, **8** has been proposed as the immediate precursor of **1** though the step involving ichangin (Figure 3) remains hypothetical [8].

Isoobacunoic (or iso-obacunoic) acid (**9**) is the constitutional isomer of **4** and **29**. Its semisystematic name is 19-deoxylimonoic acid δ-lactone. In the last decade, **9** was also isolated from the root bark of *Dictamnus angustifolius* [90,122,123] and structurally, isoobacunoic acid (**9**) to deacetylnomilinic acid (**28**) is the same as **1** is to **8**. Indeed, it was once considered as an intermediate between obacunoic acid (**29**) and **1** but labelled isoobacunoic acid has never been shown to be converted to **1**.

Deoxylimonin (or desoxylimonin) (**10**) may also named 14,15-deepoxy-14,15-didehydrolimonoic acid, di-δ-lactone. As previously discussed, **10** is generally less interesting than its epoxidised analogue **1**. A few reports on the biological activities published in the last 10 years (Table 2) will be reviewed in Section 4.

#### 2.2.6. Newly Identified Limonin Related Limonoids

**Figure 12:**
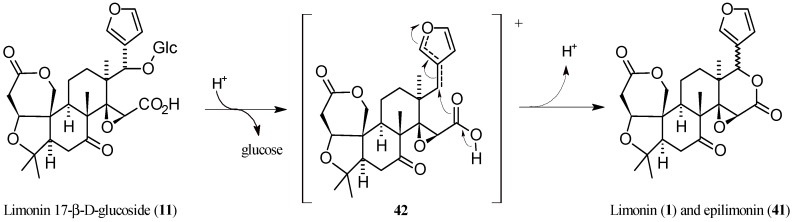
Formation of limonin (**1**) and its epimer epilimonin (**41**) from limonin 17β-d-glucopiranoside (**11**) via a furan-3-ylidene cationic intermediate (**42**); Glc = β-d-glucopyranosyl.

In 2008, two research groups separately reported on the isolation and identification of the *C*-17 limonin (**1**) epimer, named epilimonin (**41**), which displays the furan ring at *C*-17 in a β-orientation rather than the α-orientation as in **1** [10,142]. In particular, Glabasnia and Hofmann [10] investigated the hydrolytic liberation of **1** from its precursor limonin 17β-d-glucopyranoside (**11**) in orange juice samples. When the aqueous glucoside solution was adjusted to pH 1.5 and incubated for 4 h at 60 °C, a new compound was isolated and purified by semi-preparative high pressure liquid chromatography (HPLC), and its chemical structure was determined by tandem mass spectrometry (MS-MS) and 1D/2D-NMR experiments. The Authors concluded that **41** was unequivocally identified for the first time. A reaction pathway showing the formation of the two epimers from (**11**), favoured by *C*-17 furan ring through anchimeric assistance was also proposed (Figure 12) [10]. In 2003, while studying the human bioavailability of limonoid glucosides, a metabolite with similar chromatographic behavior and mass spectrum as **1** was identified, but it was not characterized at that time [143]. In 2008, the unknown compound was isolated by fractional crystallization monitoring each step by means of HPLC coupled to photodiode array and evaporative light scattering detectors (HPLC-PDA-ELSD). This metabolite was finally identified as the *C*-17 epimer of **1** through side-by-side comparison of the corresponding physical properties, including MS, IR, ^1^H- and ^13^C-NMR [142].

**Figure 13:**
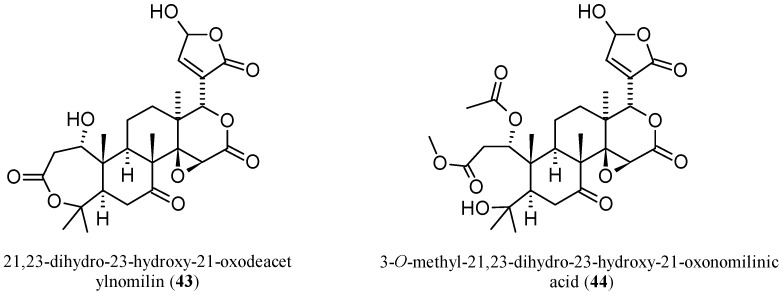
Structures of two new pseudoacids isolated from *Citrus sudachi*.

Three new pseudoacids were identified in the last decade, including two nomilin- and one ichangin-related limonoids. By extraction from *C. sudachi* peels and separation through HPLC, 21,23-dihydro-23-hydroxy-21-oxodeacetylnomilin (**43**, Figure 13) and 3-*O*-methyl-21,23-dihydro-23-hydroxy-21-oxonomilinic acid (**44**) were identified by spectroscopic (IR, ^1^H-NMR, ^13^C-NMR, heteronuclear multiple bond correlation (HMBC), nuclear Overhauser effect spectroscopy (NOESY) and spectrometric (high resolution fast-atom bombardment mass spectrometry (HR-FAB-MS) analyses. However, as suggested by the authors, it is possible that **44** is an artefactual derivative of **43** that might be formed during the extraction and purification processes [6].

**Figure 14:**
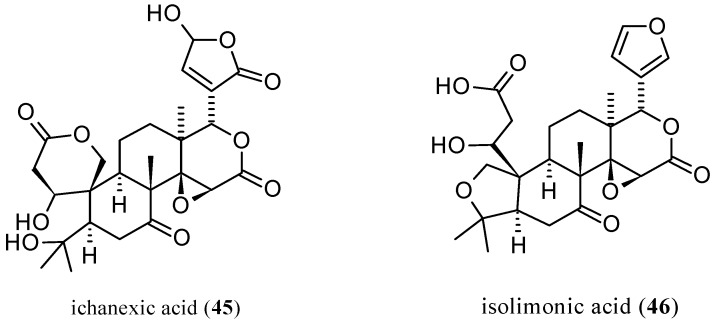
Structures of ichanexic acid (**45**) and isolimonic acid (**46**).

The third new pseudoacid, ichanexic acid (**45**, Figure 14), was identified in sour orange (*C. aurantium* L.) together with isolimonic acid (**46**), with the latter being isolated for the first time in its native form and not as the methyl ester. Ichanexic acid (**45**) is structurally related to ichangin (**8**) as isolimonexic acid (**6**) is to **1**. Isolimonic (or isolimonoic acid, **46**) is a constitutional isomer of **8**. The purity of the isolated compounds were analyzed by TLC and HPLC and their structures were identified by one-dimensional (^1^H-, ^13^C-) and two-dimensional (^1^H-H and ^1^H-^3^C) NMR experiments. Axial configuration was assumed for *C*-18 methyl group and the stereochemistry of endocyclic methine and methylene protons were assigned by nuclear Overhauser enhancement (NOE) experiments supporting the proposed structures [144]. 

In the final analysis, it is worth reviewing some rather puzzling new limonoids such as the (*E*)-isomer of obacunoic acid (**29**) which was recently isolated from the root bark of *Dictamnus angustifolius* [122]. The geometry of the double bond was established on the basis of the vinylic protons’ coupling constant in the ^1^H-NMR analysis vis-a-vis those previously reported for the the β-d-glucoside of *trans*-obacunoic acid [145]. Hence, **29** was a novel limonoid named by Sun and co-workers as dictangustone A [122]. In a recent review article [90], limonoic acid (**31**) was erroneously reported as a limonoid isolated from *Dictamnus dasycarpus* although the original studies presented it as a neutral limonoid isomeric to limonin (**1**) [127,146]. Another recent review on the antioxidant activity of CLs has reported “millington acid 17-β-d-glucoside (NAG)” and “deacetylation millington acid 17-β-d-glucoside (DNAG)” as limonoids [31]. Indeed, “(downtown) milligton acid” can be found in some Internet pages to indicate nomilinic acid (**30**) but, despite our efforts, we could not find how this curios name was conceived and given to **30**.

## 3. Isolation and Identification of Limonoids from the *Citrus* Genus

In the last decade, several articles have been published on the isolation and identification of limonoids from various *C. species*. A wide spectrum of analytical methods were used, including the well-known thin layer chromatography (TLC), nuclear magnetic resonance (NMR), mass spectrometry (MS), high pressure liquid chromatography (HPLC), and capillary electrophoresis (CE) techniques. Advanced complementary analytical methods included tandem MS (MS/MS), liquid chromatography/electrospray ionization MS (LC-ESI-MS), atmospheric pressure chemical ionization MS (APCI-MS), and collisionally activated dissociation (CAD) MS/MS. These techniques have been well reviewed in the earlier literature [1,36,147,148,149]. In this section, we focus on the isolation and identification of both new and known limonoids by using improved techniques and/or from varieties of *Citrus* never investigated until that time. Moreover, attention is paid to metabolomic studies and investigation on secondary metabolite pathways. For the isolation and identification of limonoids from *C.* species by using conventional techniques, the reader may refer to other useful reports [4,116,117,150].

### 3.1. Limonoid Aglycones

Several studies were aimed at the identification of limonoid aglycones in *Citrus* fruits as they are known to be responsible for the gradual bitterness observed in citrus juices. The limonin (**1**) content of the Iranian orange juice concentrates (OJCs), for example, was determined by reversed-phase HPLC and spectrophotometric analysis. With regards to the HPLC analysis, the reconstituted OJC samples were injected into a column with only simple filtration through a nylon filter without extraction. Both methods showed that most samples contained high amounts of (**1**), which implies that the Iranian local orange cultivars (i.e., *rasmiye shomaal*) should be categorized in the bitter oranges group [151]. Through a combination of HPLC and ESI/MS, as many as 11 limonoid aglycones have been isolated and identified in the fruit peel and seeds of *C. pyriformis* Hassk. In particular, **1** and deacetylnomilin (**4**) have been isolated from fruit peel, whilst nomilin (**2**) and ichangin (**8**) were obtained from defatted seeds after chromatographic separation. The isolated compounds were identified by MS (electron ionization (EI), chemical ionization (CI and ESI), 1D- and 2D-NMR experiments (attached proton test (APT), correlation spectroscopy (COSY), heteronuclear single-quantum correlation (HSQC), HMBC, and NOESY) and comparison with literature data as well as authentic substances. Quantitative and qualitative analysis of CLs from *C. pyriformis* were also determined by using LC-ESI/MS leading to the tentative structural assignements of seven more aglycones [3]. Furthermore, while comparing the chemical profiles of *C. wilsonii* Tanaka (CWT) and *C. medica* L. (CML), **1**, **2**, and obacunone (**3**) were unequivocally characterized among 25 compounds identified through TLC and HPLC-quadrupole time-of-flight-QTOF-MS. The quantitative results obtained by the HPLC coupled with diode array detector (HPLC-DAD) method demonstrated that (**2**) was the most dominant constituent in CML and also indicated that there were significant differences in chemical composition between the two species [152]. In a study dedicated to the simultaneous quantification of coumarins, flavonoids, and limonoids in Fructus Citri Sarcodactylis by HPLC-DAD, **1** and **2** were identified among the 11 major bioactive components [153]. With the aim of screening new sources of **1** and **2** among *C. species*, reversed-phase HPLC analyses were also performed on different citrus cultivar and, in some cases, on different fruit tissues [65,154,155,156].

**Figure 15:**
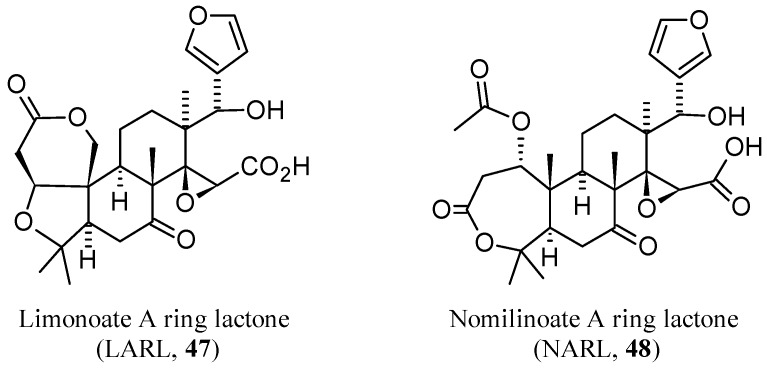
Structures of limonoate A-ring lactone (LARL, **47**) and nomilinoate A-ring lactone (NARL, **48**).

Only two studies reported the isolation of limonexic acid (**5**) in the last decade (two further studies reporting on the isolation of both **5** and its β-d-glucoside are treated in Section 3.3 [48,52]. After isolation from *C. aurantium* var. *amara*, **5** was identified through MS and NMR spectra [28]. It was also isolated from the stem bark of *C. medica* L. var. *sarcodactylis* SWINGLE, together with **1** and **2** which were also isolated from the root bark, and identified by comparison of their spectroscopic data (UV, IR, NMR, and MS) with those reported in the literature [25]. Lastly, a method has been reported for the identification of limonoid A ring lactones in citrus samples. In fact, since only indirect chemical and biochemical techniques [157,158,159] or MS detection were available, Breksa III et al. finally developed a rapid and reliable LC-ESI-MS method for the direct quantification of limonoate A Rring lactone (LARL, **47**, Figure 15) and nomilinoate A ring lactone (NARL, **48**, Figure 15) in a wide variety of citrus juices. These limonoid A-ring lactones were isolated by solid-phase extraction from juice samples and analyzed by negative ion LC-ESI-MS while their concentrations were established by fluorescence spectroscopy [160].

### 3.2. Limonoid Glucosides

**Figure 16:**
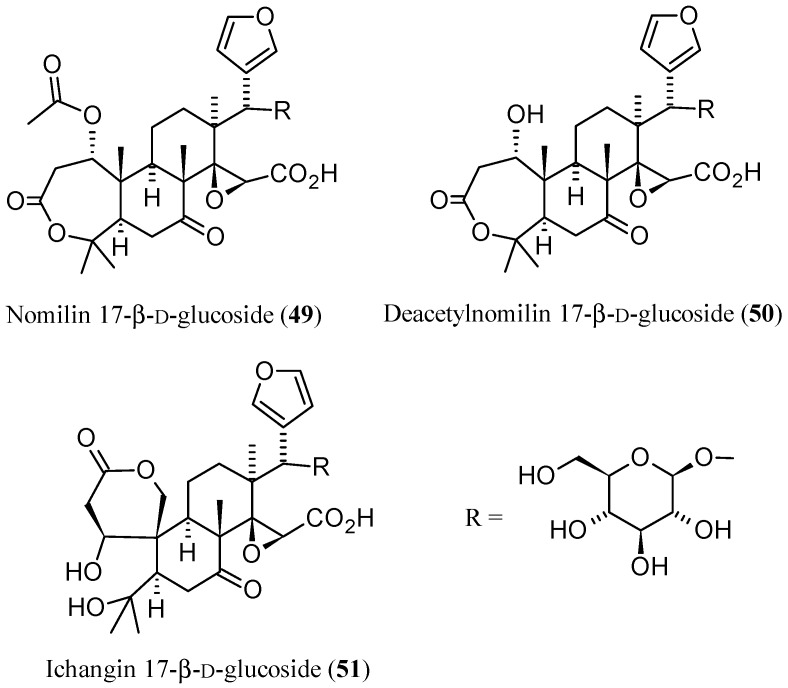
Structures of limonoid glucosides isolated from *C. species* through recently developed methodologies.

Rapid and simple separation methodologies are essential to obtain pure citrus limonoid glucosides for biological evaluations [161]. On the other hand, until the last decade, no method of separation for aglycones from glucosides was available [162]. In this regard, two studies reported on a reversed-phase flash chromatography technique developed for the separation and isolation of limonoid glucosides whose identities were then confirmed by a suitable spectrometric method. First, Raman et al. [161] purified several limonoid glucosides extracted from defatted seed powder of grapefruit (*C. paradisi* Macf.) by reversed-phase flash chromatography. The procedure yielded two glucosides with purity higher than 90% which were subsequently identified by ESI-MS as nomilin 17-β-d-glucopyranoside (**49**, Figure 16) and nomilinic acid 17-β-d-glucopyranoside (**14**) [161]. Four years later, a decigram-scale method was developed by Breska III et al. for the separation of limonin 17-β-d-glucopyranoside (**11**) from contaminating bitter **1** through C18 flash chromatography. The identities of the two limonoids were then confirmed by LC-MS analysis [163]. With regards to their identification, a rapid and selective LC-MS method has been applied to characterize CLs in citrus juices, extracts, and partially purified liquid samples, and estimate their relative concentrations without the need to treat or dilute the samples. A phenyl stationary phase, as an alternative to C18 was employed in most of the HPLC and LC-MS analysis undertaken [162].

### 3.3. Limonoid Aglycones and Glucosides

Despite the number of analytical methods reported for either CL aglycones or glucosides quantification, until 2007, there was no method for the simultaneous quantification of CL aglycones and glicosides in citrus fruits and seeds. Vikram et al. finally developed a reversed-phase HPLC method coupled with DAD for their simultaneous quantification. By using a C18 column and a binary solvent system (3 mM phosphoric acid/acetonitrile), five limonoid aglycones (**1**, **2**, **8**, **9**, and **46**) and two glucosides (**11** and **13**) were simultaneously identified in four varieties of citrus fruits and seeds, namely Rio Red Grapefruit, navel orange, valencia orange, and tangerines. Determining the limonoid concentrations directly from the peak area, **1** and **11** were found to be the predominant limonoid aglycone and glucoside, respectively, in all the tested samples [164]. By using the same method, three limonoid aglycones (**8**, **9**, and **46**) and two limonoid glucosides (**13** and **15**) were purified from sour orange (*C. aurantium* L.) seeds. This time, the identities of the purified compounds were also confirmed by positive APCI-ESI-MS spectra for aglycones and negative ion APCI for glucosides and the spectra were compared with published data. It is noteworthy that a cation H^+^ exchange column was employed to separate limonoids from flavonoids [83]. Moreover, the soft ionization technique (CID) was coupled with the above described reversed-phase HPLC, and limonoid aglycones and glucosides were simultaneously identified from complex citrus samples. In particular, **1**, **2**, **4**, **8**, and **46** were identified by positive ion CID MS/MS, whereas the glucosides **11**–**15** were identified by negative ion CID MS [165]. On the other hand, RP-HPLC-PDA-MS was used for the qualitative and quantitative characterization of limonoid aglycones and glucosides in juice, peels, pulps, and seeds of two bergamot cultivars (*Fantastico* and *Femminello*). Limonoid aglycones were the most abundant in seeds and peels (70% and 80% of the total, respectively), while limonoid glucosides were predominant in juices and pulps (61% and 76% of the total, respectively), thus reflecting their corresponding lipophilicity. Calibration curves were built by using pure limonoids isolated from bergamot seeds and juice through 2D-HPLC/PDA/MS preparative system [5].

A simple and rapid colorimetric method, amenable to the simultaneous analysis of multiple sample with a plate reader, has been proposed by Breksa III et al. [166] for estimating the total **1** and **11** concentrations in citrus juices. Until that time, no methods for the spectrophotometric determination of limonoid glucosides were known, since the above-reported Abbasi’s spectrophotometric method is specific for limonin aglycone [151]. The new method is based on the formation of red to orange colored derivatives resulting from the treatment of **1**, **11**, or a fruit extract with 4-(dimethylamino)benzaldehyde (DMAB) in the presence of perchloric and acetic acids. Limonoate A-ring lactone (LARL, **47**) was also tested and found to mirror the properties of **1**, likely due to the rapid conversion of **47** into **1** when treated with the acidic indicator solution [166]. By using this colorimetric method, the first analysis of total limonoid content in sour orange juices was also performed.

CL glucosides, **47**, and **48** were also analyzed by LC-MS as previously reported [160]. The results obtained suggest that sour oranges at maturity are not distinctly different from their sweet orange counterparts in terms of LARL and NARL concentrations, and therefore may share common genomic sequences that encode for the limonoid biosynthetic pathways. On the contrary, there is a significant variability within and across *C. species* with regard to their total limonoid glucosides concentrations which are not entirely dependent upon genetic background. Indeed, environment factors such as location, plant health, cultivation practices, and fruit maturity are contributing factors for limonoids variability in concentrations. In general, when compared to other citrus juices, the limonoid glucoside content in the sour orange juices is much lower than that of sweet oranges and, among the limonoid glucosides present, nomilin related glucosides, **13**, **14**, and deacetylnomilin 17-β-d-glucoside (**50**), were predominant [167]. Finally, in a project aimed at using renewable natural resources, Minamisawa et al. [168] proposed in 2014 the extraction of large amounts of limonoids from waste yuzu (*C. junos*) seeds. By using reversed-phase HPLC and LC-MS, four limonoid aglycones (**1**–**4**) and seven limonoid glucosides; **11**–**14**, **49**, **50**, and ichangin glucoside (**51**, Figure 16) of the limonin group were identified from the yuzu seed extracts. Their amounts were found to be higher than those found in other citrus fruits [168]. The isolation and identification of both limonoids aglycones and glucosides by routinary HPLC and spectrometric or spectroscopic analyses is reported in several recent papers [48,52,169].

### 3.4. Metabolomic Analysis

Metabolic profiling has become an invaluable tool to identify as many metabolites as possible in biological systems. It is well known that metabolites are the downstream products of gene expression and, as such, subjected to a thorough selection process [170]. Therefore, secondary metabolites can be used either as quality traits or as markers for the selection and/or certification of different fruit sources [171] or for the physiological evaluation of plant genotypes [172]. In 2014, two different MS ionization methods, direct analysis in real time MS (DART-MS) and HPLC-ESI-MS, were explored by Pan et al. [172] to profile the metabolites in the fruit flesh of “Anliu” sweet orange and its bud mutant ‘Hong Anliu’. A total of 133 metabolites were tentatively identified, representing thus by far the most comprehensive metabolomic analysis in citrus. Among them, four limonoids were identified: **1**, **2**, **3**, and **8**. In general, more ions were detected in the LC-ESI-MS experiments, clearly indicating that the matrix effects play an important role in the DART measurements. Conversely, the major advantage of the DART-MS is in that this technique can perform sensitive and comprehensive analyses for metabolites in intact biological samples without the need of any sample preparation. This study provided a comprehensive assessment of metabolites in orange fruits and also revealed metabolomics differences in fruits between two isogenic orange genotypes [172].

Limonin with 84 of its metabolites were also identified in a polar extract from lemon (*C. limon*) by liquid chromatography-quadrupole time of flight-tandem mass spectrometry (LC-QTOF-MS) that provided high mass resolution of the parent ion and their fragments for each metabolite. Thus, the tentative identification of all the compounds was based on MS/MS spectra by comparison with databases and without reference standards [173]. A reversed-phase liquid chromatography method coupled to a QTOF-MS was developed to analyze the metabolite profiles of juices from 12 commercial varieties grouped into blonde and navel types, mandarins, lemons, and grapefruits. Several limonoids were identified by analysis of such mass spectra and it has been demonstrated that all orange and grapefruit varieties showed high contents of **1** and **11**. The rest of the identified limonoids were highly abundant in oranges and, in particular, Sucrenya cultivar showed a specific accumulation of **3** and **47** [170]. Similarly, non-targeted HPLC-ESI-QTOF-MS based metabolomic analysis was performed by Wang et al. [2] to study more thoroughly than before the tissue-specific metabolism in citrus. Particularly, four fruit tissues (flavedo, albedo, segment membrane, and juice sacs) and different *C. species* (lemon, pumello and grapefruit, sweet orange and mandarin) were investigated. More than 54 metabolites, including **1** and **2**, were putatively identified and a differential accumulation patterns of them in various tissues and species was revealed. In particular, **1** and **2** were mainly found in the segment membrane; the lowest levels were detected in flavedo. Furthermore, lemon showed the highest abundance of both bitter metabolites, while the least accumulation was observed in orange [2].

A simple and rapid ultra-performance liquid chromatography coupled with quadrupole time-of-flight mass spectrometry method (UPLC-Q-TOF-MS) was applied to assess the chemical compositions of *C. reticulata* Blanco cv. Ponkan for the first time. According to the chromatography retention time, UV spectra, exact molecular weight, and high energy fragment ions combined with the information of reference standards or literature, a total of 32 bioactive compounds were unambiguously identified or tentatively characterized in Ponkan peel methanol extract. The compounds included **1**, **2**, **14**, **46**. Furthermore, **14** and **46** were characterized for the first time in Ponkan peel. It is noteworthy that the method is very fast since the well separation of chemical components in Ponkan peel was completed in 7 min [7].

### 3.5. Metabolic Transformations of Secondary Metabolites

**Figure 17:**
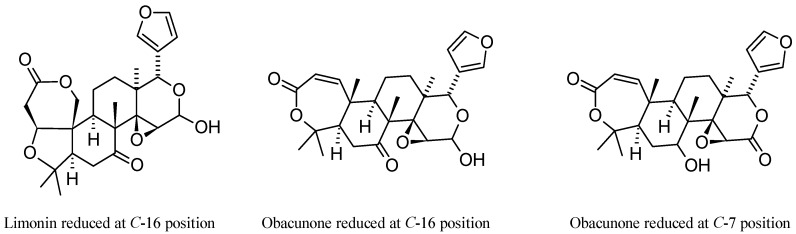
Structures of limonin (**1**); and obacunone (**3**) metabolites identified in liver microsomes.

In 2014, Ren et al. [174] carried out a comparative investigation on the metabolism of **1** and **3** in five different species of liver microsomes (human, monkey, dog, rat, and mouse liver microsomes) and in zebrafish through ultra-high performance liquid chromatography (UHPLC) coupled with a high-resolution LTQ Orbitrap mass spectrometer. The ESI-HR-MS/MS fragmentation behaviors of **1** and **3** were investigated for the first time and their metabolic rates were estimated using UHPLC/HRMS. The results of this study demonstrated that the two parent compounds present similar metabolic processes, with a reduction reaction being the major metabolic pathway for both CLs. Based on the accurate MS/MS spectra and proposed MS/MS fragmentation pathways, the chemical structures of three metabolites were identified: one metabolite of **1** reduced at *C*-16 position and two metabolites of **3** reduced at *C*-16 or *C*-7 positions, respectively (Figure 17). The *C*-16 reduced metabolites of both **1** and **3** were only detected in liver microsomes while the *C*-7 reduced metabolite of **3** was found in both liver microsomes and zebrafish incubation systems. This is probably caused by metabolic differences between mammals and fish or, more simply, **1** probably cannot be absorbed in zebrafish (cf. cLog *P* values for **1** and **3** in Table 2) [174]. One year later, a more exhaustive study in human liver microsomes, performed on **2**, revealed other metabolites arising from isomerization, hydroxylation, and conjugation with glycine of reduced limonoids, with the latter metabolic pathway being observed for the first time. It has been demonstrated that the cytochrome P450 (CYP) mainly involved were CYP2D6 and CYP3A4 since they play an important role in the isomerization and glycination reaction in human liver microsomes. Other CYP isoforms were considerably less active. For the sake of completeness, we point out here that in the article related to this study, a metabolite named “nominin” is reported which is not mentioned nowhere else. On the basis of the reported structure, we can state that it was the well-known CL nomilin (**2**) [175].

Tian et al. [176] studied for the first time the metabolism of **11** in culture media by HPLC. In particular, four microorganisms widely present in the human lower gastrointestinal tract (*Enterococcus fecalis*, *Escherichia coli*, *Lactobacillus salivarius*, and *Candida albicans*) were investigated since limonoid glucosides, as already known for flavonoid glucosides [143], could be hydrolyzed by microorganisms commonly found in the colon and then absorbed in their aglycone forms. After the removal of interfering substances via passing the culture medium through an octadecylsilane (ODS) cartridge then eluted with suitable solvent, the sample was injected into the HPLC apparatus and the concentration of **11** and its possible metabolites were determined. ESI-MS was used to confirm the identity of **11**. Significant metabolic activity of *E. coli* and *C. albicans* on **11** was observed and, although **1** was not detected, several unidentified metabolites were found suggesting that **11** may be metabolized by intestinal microorganisms. A possible explanation for the absence of **1** in the medium of all samples is that it could be in turn converted into other metabolites by the gut microorganisms.

## 4. Pharmacological Properties

In this section, the main biological properties of CLs of the limonin (**1**) groups studied in the last decade are examined (see also Table 2). Particular emphasis is given to anticancer activity studies. As free radicals and/or reactive oxygen species are known to be associated with tumorigenesis, the cytotoxic activity of CLs is usually studied together with their antioxidant properties. Hence, both activities are examined together while other effects including antimicrobial and insecticidal activities among others are presented separately.

### 4.1. Anticancer and Antioxidant Activities

#### 4.1.1. In Vitro Tests

The over-expression of cytochrome P450 (CYP) isoenzymes such as CYP1A2, CYP1B1, CYP19 and CYP3A4 has been specifically implicated in the onset of cancers of the lung, breast, colon and prostate. Thus, Poulose et al. [47] have evaluated the effects of limonoid aglycones and glucosides on the activity of human CYP isoenzymes including CYP1A2, CYP1B1, CYP19, and CYP3A4. Individual limonoids were tested for O-dealkylase and hydroxylase activities of the human CYP isoenzymes using ethoxyresorufin, methoxyresorufin and dibenzylfluorescein as substrates. Significant partial to high inhibition of CYPs was observed in a dose-dependent manner at concentration higher than 2 μmol. Kinetic analyses further indicated that limonin glucoside (**11**) inhibited CYP19 competitively (IC_50_ 7.1 μM), whereas nomilinic acid glucoside (**14**) inhibited it in a noncompetitive manner (IC_50_ 9.4 μM). The differential inhibition of CYPs can be ascribed to structural variations of the limonoid nucleus. Similar results were documented by Han et al. [53] who showed that limonin (**1**) inhibits CYP3A4 isoenzyme in human liver microsomes. LC-tandem mass spectrometry was used and IC_50_ values of 6.20 μM and 19.10 μM were found against CYP3A4 enzymatic activity with testosterone and midazolam as substrate, respectively.

The antiproliferative activity of **1** was verified by the analysis of mitochondrial membrane potential and intracellular calcium accumulation in human colon adenocarcinoma (SW480) cells [57]. A certain additivity with the antiproliferative activity of curcumin was noted and the Authors concluded that consumption of curcumin and limonoids together may offer greater protection against colon cancer. Langeswaran et al. [55] also studied the antiproliferative properties of **1** against human hepatoma HepG2 cells by employing lactate dehydrogenase (LDH) cell viability assay. Induction of apoptosis in HepG2 cells by **1** was evidenced by western blot analysis of Bax, cyclin D1, caspase-3, and caspase-9. Limonin (**1**) was also found in this study to downregulate the expression of LRP5, LRP6, and DKK wnt players, which forms a rationale for further investigation on effect of **1** cancer therapy. Das et al. [56] further showed that the treatments of IOMM-Lee and CH157MN meningioma cells with (**1**) can induce apoptosis with enhanced phosphorylation of glycogen synthase kinase 3 β (GSK3β) via inhibition of the Wnt5/β-catenin pathway (EC_50_ = 25 μM). While studying the effects of **1** on intestinal polyp from Apc-mutant Min mice, Shimizu et al. [54] have observed a reduction of neoplastic cell proliferation after treatment with **1,** along with reduced level of expression of c-Myc and MCP-1 mRNA in the polyp part. Moreover, they found that **1** also significantly inhibited T-cell factor/lymphocyte enhancer factor-dependent transcriptional activity in a dose-dependent manner in the Caco-2 human colon cancer cell line.

El-Readi et al. investigated the P-gp reversal activities of **1** and deacetylnomilin (**4**), isolated from *C. jambhiri* and *C. pyriformis* in human leukemia cells (CEM/ADR5000), and also their potential cytotoxicity against this cell line, as well as its parental cell line CCRF-CEM (Adriamycin-sensitive human leukemia cell line, no expression of P-gp) and Caco-2 cells [51]. In this study, limonin (**1**) was shown to inhibit the efflux of the P-gp substrate rhodamine 123 in a concentration-dependent manner. Limonin (**1**) as the most potent P-gp inhibitor and in its non-toxic concentration (20 μM) significantly enhanced the doxorubicin cytotoxicity 2.98-fold (*p* < 0.001) and 2.2-fold (*p* < 0.001) in Caco2 and CEM/ADR5000 cells, respectively. The IC_50_ values found for **1** in wild-type CCRF-CEM, multidrug-resistant CEM/ADR5000, and Caco-2 cells were 520 μM, 285 μM, 159 μM, respectively. Limonin (**1**), limonexic acid (**5**), and isolimonexic acid (**6**), isolated from Mexican lime (*C. aurantifolia*) juice also showed cytotoxic activity against the pancreatic cancer cells Panc-28 [48]. The methanolic extract of this plant exhibited the maximum activity with an IC_50_ value of 81.20 μg/mL after 72 h. The reported inhibition of Panc-28 cells was in the range of 73%–89%, at 100 μg/mL after 96 h. The involvement of apoptosis in induction of cytotoxicity was further confirmed by expression of Bax, Bcl-2, caspase-3, and p53 [48]. Similarly, Patil et al. [52] studied the proliferation inhibitory activity in human pancreatic (Panc-28) cell lines by **1**, limonin glucoside (**11**), **5** and **6** isolated from *C. aurantifolia*. The IC_50_ values obtained through the MTT assay of purified compounds were >100 μM (24 h); 49.84 μM (48 h); 42.40 μM (72 h).

Murthy et al. showed that obacunone (**3**) and its glucoside (**12**) inhibited colon cancer (SW480) cell proliferation with IC_50_ values of 97 and 109.7 μM respectively, at 24 h [73]. Sequence of events such as decreased ratio of bcl2/bax gene transcription, activation of caspase-3, and fragmentation of DNA in cells treated with both compounds demonstrated induction of apoptosis by limonoids. Additionally, higher induction of cytochrome-c in the cytosol suggests the activation of the intrinsic apoptosis pathway by limonoids. The involvement of apoptosis was also confirmed through expression of bax, bcl2, pro-caspase-3 and caspase-9. More eover, both CLs activated p21 and arrested cells at G1 and G2/M phase. The cytotoxic effect of **3** was also tested on human breast cancer (MCF-7) and non-malignant (MCF-12F) breast cells [74]. Through the MTT assay, **3** has been shown to strongly inhibit MCF-7 cell proliferation without affecting non-malignant breast cells. Treatment with **3** appeared to increase apoptosis by upregulating the expression of pro-apoptotic protein, Bax, and down-regulating the anti-apoptotic protein Bcl2, as well as inducing G1 cell cycle arrest. In addition, **3** significantly inhibited aromatase activity in enzyme inhibition assay. Finally, the exposure of MCF-7 breast cancer cells to **3** down-regulated the expression of inflammatory molecules including nuclear factor-kappa B (NF-κB) and cyclooxygenase-2 (COX-2). The IC_50_ of **3** for aromatase inhibition was 28.04 μM [74]. Compounds **3** and **12** were also shown to inhibit the proliferation of human androgen-dependent prostate cancer LNCaP cells in a dose-dependent fashion [73]. Both CLs resulted in more than 65% (*p* < 0.05) inhibition of proliferation at 100 μM and showed no further significant increase at 200 μM. On the other hand, both CLs did not show toxicity to prostate epithelial cells up to the concentration of 200 μM in 48 h treatment period suggesting they may not affect the viability of normal prostate cells. 

Limonexic acid (**5**) was tested for potential activity on human colon cancer cell (HT-29) proliferation and apoptosis. Cell proliferation, arrest of the cell cycle, and induction of apoptosis were assessed by the MTT assay, flow cytometry, and nuclear staining methods, respectively. The data indicated that **5** exerts significant cell proliferation inhibition at 5–40 μM concentrations [87]. The effects of **5** have also been studied on SW480 human colon adenocarcinoma cells where cell viability was evaluated by the MTT assay [81]. Limonexic acid (**5**) showed low but significant inhibition of SW480 cell proliferation at 50 μM (11.08%, *p* < 0.05). The antitumour activity of **5** was also investigated by testing its inhibitory effects on the proliferation and morphological variation of Hela, SMCC-7721, and B16 cell lines. The results showed that **5** had significant concentration-dependent inhibitory effects on the proliferation and morphological variation of all the three cell lines [28].

In the last decade, isolimonic acid (**46**) has been shown to be the most potent anticancer CLs. Isolated from *C. aurantium*, **46** caused the inhibition of human colon cancer cells (HT-29) proliferation within 24 h of treatment at concentrations as low as 5.0 *μ*M. The increase of G2/M stage cells, after treatment with **46**, indicates cell cycle arrest as possible mechanism [143].

Extracts from lemon seeds containing a high percentage of glucosides (e.g., **11** and **12**) have been investigated for their radical scavenging activity and apoptotic effects in human breast adenocarcinoma (MCF-7) cells and non-malignant breast (MCF-12F) cells. The highest radical scavenging activity and inhibition of MCF-7 cells in the MTT assay was exhibited by the methanol:water (80:20) extract which contained a high percentage of glucosides [86]. Poulose et al. [89] have also studied the anticancer and antioxidant activities of different CLs: **11**, **12**, deacetylnomilinic acid 17-β-d-glucopyranoside (**13**), and nomilinic acid 17-β-d-glucopyranoside (**14**). These compounds were tested for superoxide radical (O_2_^−^)-quenching activity and cytotoxic action against undifferentiated human SH-SY5Y neuroblastoma cells in vitro. All compounds scavenged O_2_^−^ as measured by inhibition of pyrogallol decomposition in a spectrophotometric assay. Quenching by **14** in particular emulated an equivalent concentration of vitamin C. Cytotoxicity was related to a concentration- and time-dependent increase in caspase-3/7 activity, suggesting that CLs could induce apoptosis [89].

Finally, Hamdan et al. evaluated the antioxidant activity of different extracts from the fresh peel of *C. jambhiri* by measuring their DPPH radical-scavenging effects [4]. They found that the chloroform fraction (reach in **1** and **2**) exerted the strongest DPPH free radical scavenging activity (IC_50_ = 119.4 ± 0.8 μM). In the past, discrepancies in antioxidant capacity measurements for CLs were reported and the possibility of contribution by impurities other the major components in the tested samples should be taken into account [1]. On the other hand, CL structures are not optimised to provide direct radical-scavenging activity as they lack aromatic and phenolic structures.

#### 4.1.2. In Vivo Tests

The effects of orally given limonin (**1**), nomilin (**2**) and aglycone mixtures on various tissues of mice have been previously investigated [59]. When three doses of 20 mg of CLs were given to 10-week-old female A/JOlaHsd mice over a period of one week, the aglycones were found to be more effective inducers of GST activity in the stomach than their respective glucosides. Vanamala et al. have also evaluated the effect of **1** against azoxymethane (AOM)-induced aberrant crypt foci (ACF) formation in Male Sprague-Dawley rats [50]. The data revealed that apoptotic index in AOM-injected rats was greatly enhanced with grapefruit and **1**. Hence, consumption of grapefruit or **1** may help suppress colon cancer development.

Following their studies in vitro [49], Perez et al. also evaluated how CLs **2**, deacetylnomilin (**4**), isoobacunoic acid (**9**), and a mixture of limonoids would influence phase II enzyme activity in female A/J mice [70]. The highest induction of GST against 1-chloro-2,4-dinitrobenzene (CDNB) was observed in stomach (whole), with the most active CL being **2** (58%), followed by **9** (25%), and **4** (19%). Deacetyl nomilin (**4**) significantly reduced GST activity against CDNB both in the intestine (small) and liver. Additionally, **9** and the limonoid mixture caused a significant reduction of GST activity against CDNB in liver. Nomilin (**2**) significantly induced GST activity against 4-nitroquinoline 1-oxide (4NQO) both in intestine (280%) and stomach (75%) while **4** showed significant induction only in intestine (73%). Induction of GST activity was also observed in intestine (93%) and stomach (45%) treated with the limonoid mixture. Finally, a significant induction of NAD(P)H: quinone reductase (QR) activity was observed after treatment with the limonoid mixture in stomach (200%). In addition, the group treated with **4** displayed an increase in QR activity in liver (183%) and intestine (22%). Even though the reported antioxidant and potential anticancer effects of CLs are promising, readers should note the following:

- While there is no doubt that antioxidant activity has been demonstrated for CLs, the reported higher micromolar concentration range is not making these group of compounds as optimal leads for this biological activity. For example, our decades or research in this field has shown that promising antioxidants should act at micro/submicromlar ranges and numerous polyphenolic compounds such as gallic acid and flavonoid derivatives fit into this category, e.g., [177,178,179,180,181,182,183,184,185,186].
- Similarly, there is no doubt that anticancer activity has been demonstrated for the numerous CLs which generally ranges from weak to good hits. This moderate activity is in line with the general anticancer profile of various natural terpenoids that bear α,β-unsaturated functional moieties, e.g., [183,187,188,189]. As there are numerous examples of other natural products that act in nanomolar ranges, e.g., [190], further medicinal chemistry studies, in addition to the one presented in Section 2, focusing on lead optimisation is necessary.
- Irrespective of drug lead identification and rather on the basis that CLs are consumed in large amount on daily basis, however, the reported cytotoxic and antioxidant activity have significant implication on the potential health/medical benefit of citrus juices. In this regard, some in vivo data already substantiated the potential benefit of CLs as anticancer agents. On this basis, there should be an urge to enhance the potency and/or increased multifunctionality (e.g., through increased antioxidant effects) of CLs.

### 4.2. Selective Toxicity against Pathogens

#### 4.2.1. Antiviral Activities

Balestrieri et al. studied the effects of *C. bergamia* (BSext) extract and two isolates (limonin (**1**) and nomilin (**2**)) on the oncogenic, delta retrovirus human T-cell leukaemia/lymphoma virus type 1 (HTLV-1) [67]. Their results showed that the efficacy of both BSext and **1** in inhibiting HTLV-1 as well as HIV-1 expression in infected cells was close to those of the effective reference standard compounds. Furthermore, the protective effects of BSext and of the purified products were shown to be associated with the inhibition of both HTLV-1 and HIV-1 real time activities in conceptually similar, cell-free assays. In another study, limonexic acid (**5**) has been shown to inhibit the expression of the hepatitis B surface antigen (HBsAg, IC_50_ = 78.05 μM) and the secretion of hepatitis B envelope antigen (HBeAg, IC_50_ = 136.0 μM) [28].

#### 4.2.2. Antibacterial Activities

Vikram et al. investigated the effect of obacunone (**3**) on the food-borne pathogen, *Salmonella enterica* serovar Typhimurium LT2, by using a cDNA microarray [76]. Transcriptomic studies indicated that **3** represses *Salmonella* pathogenicity island 1 (SPI1), the maltose transporter, and the hydrogenase operon. Furthermore, phenotypic data for the Caco-2 infection assay and maltose utilization were in agreement with microarray data suggesting repression of SPI1 and maltose transport. The same group tested the ability of ichangin (**8**), deacetylnomilinic acid glucoside (**13**) and isolimonic acid (**46**), all purified from sour orange, to inhibit cell-cell signaling and biofilm formation in *Vibrio harveyi* [83]. The three CLs demonstrated significant inhibition of autoinducer-mediated cell-cell signalling and biofilm formation. Furthermore, treatment with **8** and **46** resulted in induced expression of the response regulator gene luxO. Therefore, the ability of **8** and **46** to interfere with cell-cell signalling and biofilm formation seems to stem from the modulation of luxO expression. The results also suggest that **8** and **46** are potent modulators of bacterial cell-cell signalling. In the following study by the same authors on the inhibitory effect of several CLs on cell-to-cell communication, biofilm formation, and expression of enterohemorrhagic *E. coli* (EHEC) type three secretion system (TTSS) [77], obacunone (**3**) was found to be a potent antagonist of EHEC O157:H7 biofilm with 9%–68% inhibition in the tested concentration range (IC_50_ ≈ 117 μM). In other studies, **8** and **46** were shown to inhibit EHEC biofilm (IC_25_ = 28.3 and 19.7 μM, respectively) and adhesion to Caco-2 cells [84]. The qPCR analysis also revealed that both CLs repressed the locus of enterocyte effacement (LEE) encoded genes by ≈3 to 12 fold. In addition, flagellar master regulators (flhDC) was repressed by the two limonoids (≈3 to 7 fold) [84].

#### 4.2.3. Larvicidal and Insecticidal Activities

Looking for eco-friendly plant extracts that have potential to suppress the mosquito population, the larvicidal activity of citrus seed crude extracts was evaluated against *Aedes albopictus* by following the WHO mosquito larval bioassay methodology [68]. Among the tested citrus cultivars, Valencia Late (*C. sinensis*) was the best in terms of LC_50_ (297 ppm), % mortality (97%), and lethal time against 50% of the treated larvae (LT_50_) (18.49 h). Less efficacy was observed in freutrall early (*C. reticulate*) with LC_50_ (377.4 ppm), % mortality (88%), and LT_50_ (31 h). Using the same test, nomilin (**2**) gave lower LC_50_ (121.04 ppm) than limonin (**1**) (382.22 ppm) after 72 h of exposure. Hafeez et al. have also tested the larvicidal activity of citrus limonoids **1** and **2** against *A.* *albopictus* using the WHO methodology [69]. They found the following LC_50_ values: 305.83, 176.08, and 136.07 μM for **2** and 850.09, 600.72, and 407.09 μM for **1** after 24, 48, and 72 h, respectively.

Yu et al. evaluated the insecticidal activity of a series of esters structurally related to obacunone (**3**) against the pre-third-instar larvae of oriental armyworm (*Mythimna separata Walker*), a typical lepidopteran pest [79,80]. These derivatives where obtained by reduction of obacunone (**3**) and successive acylation of the so-obtained alcohol. Two of the **3** derivatives (*C*-7α-propionyl and *C*-7β-*n*-heptanoyl substituted congeners) were found to be more active than the parent compound. The abovementioned reported larvicidal and insecticidal effect of the few citrus limonoids is interesting given that one of the widely-used insecticidal drug of natural origin to date is azadirachtin; a limonoid isolated from neem tree (*Azadirachta indica).*

### 4.3. Other Bioactivities of Citrus Limonoids Studied in the Last Decade

#### 4.3.1. Analgesic and Anti-Inflammatory Activities

Kim et al. studied the involvement of CLs in inflammatory pathways via modulation of p38 MAP kinase activity in vascular smooth muscle cells [62]. Nomilin (**2**) exhibited the highest inhibition of p38 MAP kinase activity, followed by limonin (**1**), deacetylnomilin (**4**), and defurannomilin (**36**). Furthermore, TNF-α induced p38 MAP kinase activity in the smooth muscle cells was shown to be completely inhibited by **2**. In this regard, the design and synthesis of a series of water-soluble derivatives of limonin (**1**) as analgesic and anti-inflammatory agents are presented in Section 2.2.1 [45].

Mahmoud et al. showed that **1** promoted the attenuation of markers of hepatic damage (elevated liver enzyme activities and total bilirubin) and hepatic inflammation (TNF-α, infiltration of neutrophils), oxidative stress, and expression of TLR-4 but not TLR-2 in rat model of acute hepatic inflammation [60]. The same group described the protective effects of **1** on experimentally-induced hepatic ischemia reperfusion (I/R) injury in rats. The mechanism of these hepatoprotective effects was related to the antioxidant and anti-inflammatory potential of **1** mediated by the down regulation of TLR-signaling pathway [61].

Lu et al. investigated the protective effects of compounds extracted from the flowers of *C. aurantium* L. var. amara Engl, against carbon tetrachloride (CCl_4_)-induced hepatocyte injury. They used the human hepatic cell line HL-7702 to determine cell cytotoxicity, cell viability, levels of hepatic marker enzymes and the level of malondialdehyde (MDA) [82]. They found that limonexic acid (**5**) could significantly reverse the CCl_4_-induced suppression of HL-7702 cell viability suggesting its potential role in protection against xenotoxicant-induced liver injury. In another study, **5** showed anti-inflammatory activities at the concentration range of 12.4–199 μM by inhibiting NO production in LPS-induced RAW264.7 macrophages [28].

Kelley et al. studied the effects of limonin glucoside (**11**) on circulating biomarkers of chronic inflammatory diseases such as nonalcoholic fatty liver disease (NAFLD), diabetes, CVD, and cancer in a cross-over, placebo controlled, double-blind study in overweight/obese individuals [88]. They observed that **11** decreased the circulating level of liver enzymes γ-glutamyl transferase (33.8%), alanine aminotransferase (13.1%), and alkaline phosphatase (10.1%). Limonin glucoside (**11**) also significantly decreased the blood levels of the proinflammatory markers complement C3 (6.4%), matrix metallopeptidase 9 (MMP-9) (38.7%), and tumor necrosis factor α (TNF-α) (10.7%). To deepen the effect of **11** on immune cell functions, the same group then designed a second double-blind, randomized, cross-over study [85]. After ten overweight/obese human subjects were served purified **11** or placebo drinks for 56 days each, the percentage of CD14+CD36+ cells in whole blood was analyzed by flow cytometry. No differences were observed for CD14+CD36+ monocyte populations, T-cell proliferation, or the production of T cell and monocyte cytokines between the two treatments. Thus, it was concluded that **11** does not affect ex vivo functions of T cells and monocytes, whereas it decreased several circulating markers of hepatic inflammation. More scientific research is however need to substantiate this conclusion. In view of limonoids from other natural sources are generally known to have potent anti-inflammatory effects, e.g., [191], the above–mentioned pharmacological effects of citrus limonoids merit further investigation.

#### 4.3.2. Anti-HyperGlycemic Properties

The G protein-coupled bile acid receptor 1 (TGR5) is thought to be a promising drug target for metabolic diseases because its activation prevents obesity and hyperglycemia. Ono et al. observed that nomilin (**2**) is an activator of TGR5 in mice fed a high-fat diet (HFD) suggesting a novel biological function of **2** as anti-obesity and anti-hyperglycemic agent [71]. In order to clarify the potential role of **3** in metabolic regulation, Horiba et al. also studied the dietary supplementation of the compound on obese KKAy mice [78]. It was found that **3** stimulates the transcriptional activity of TGR5 in a dose-dependent manner. In addition, **3** inhibited adipocyte differentiation in 3T3-L1 cells and antagonized ligand-stimulated peroxisome proliferator-activated receptor γ (PPARγ) transcriptional activity [78]. 

#### 4.3.3. Inhibition of Osteoclastogenesis

Kimira et al. studied the potential use of nomilin (**2**) for the inhibition of osteoclastogenesis in vitro [72]. They measured cell viability of the mouse RAW 264.7 macrophage cell line and mouse primary bone marrow-derived macrophages (BMMs) with the Cell Counting Kit. They observed that **2** significantly decreased TRAP-positive multinucleated cell numbers (a measure of osteoclast cell numbers) when compared with the control. Moreover, the non-toxic concentrations of the compound decreased bone resorption activity and down regulated osteoclast-specific genes (NFATc1 and TRAP mRNA levels) coupled with suppression of the MAPK signaling pathway. This study therefore suggests a therapeutic potential of **2** for the prevention of bone metabolic diseases such as osteoporosis.

## 5. General Summary and Conclusions

In principle, medicinal chemistry-based studies should provide reliable data through sound design and careful biological evaluation of series of closely related compounds differing for relatively small variations. Similarly, structure-activity relationships (SAR) considerations should rely on quantitative measurements (e.g., IC_50_) accompanied by an error indicator (e.g., SEM) and the number of replicates (*n*). In order to have a fair comparative assessment of relative potencies, concentrations expressed in molar basis should be employed instead of those in gram weight quantities (e.g., μg/mL). Moreover, the activity of test compounds must be compared with one or two well-known reference standards sharing the same biological property. When synergistic behaviour is investigated, the isobole analysis should also be performed [192]. On these analysis basis, a number of studies on CLs isolated from plants in recent years approximate the above ideal drug leads picture. Nevertheless, the medicinal chemistry of CLs is still in its infancy and several problems are to be faced in order to promote its maturation. First of all, natural CLs differ for relatively marked structural variations (e.g., removal of rings and chemical functions; cf. Table 1). Secondly, the results obtained from in vitro studies show high variability, sometimes reflecting poor purity of the studied CLs [1]. Of course, synthetic analogues would solve both of the above problems but the synthetic procedures to obtain such complex structures as CLs seem to be beyond the skills of most medicinal chemists and substantially unpractical. As an example, the recently proposed virtuosistic total synthesis of racemic limonin (**1**) from geraniol takes 35 steps with overall yield as low as 0.2% [193]. Semisynthetic derivatives are obviously more accessible. Unfortunately, natural starting materials are generally obtained through long, relatively sophisticated procedures. As an example, a recently developed method includes three steps: removing most impurities by macroporous resin HZ-816, isolating limonin (**1**) by high speed counter current chromatography (HSCCC), and isolating nomilin (**2**) and isoobacunoic acid (**9**) by semi-preparative HPLC [194]. Generally, CLs are obtained at the decigram scale level [163]. Actually, only **1**, obacunone (**3**), and deoxylimonin (**10**) were used in the last decade as starting materials to obtain few related derivatives. Improved large-scale extraction methodologies and the abundantly available waste citrus materials [23,168,169,195] should allow easier access to starting materials in the future. Then, ligand efficiency metrics (LEM) guided derivatization of the latter should furnish new CL analogues to prove the reliability of CLs as defenders and promoters of human health.

While the abovementioned medicinal chemistry in general and structure-activity relationship study in particular may yield a better lead CLs in the future, researches in the last decade have already documented promising structural and pharmacological diversity for these group of compounds. The complexity of CLs with their high number of rings and oxygenation pattern endowed their tremendous capacity to design new natural CLs-based therapeutic leads. With regard to the pharmacological effects investigated so far, anticancer and antioxidant effects, though as yet at therapeutically unfavourable doses as potential drugs, have been recorded. Given that these group of compounds are readily available in our daily diet and consumed in large quantities, however, their potential therapeutic/nutraceutical value cannot be underestimated. On the other hand, the reported antimicrobial, insecticidal and potential effects against metabolic diseases further make CLs a formidable group of interesting natural products that merit further studies.

## Figures and Tables

**Table 1:**
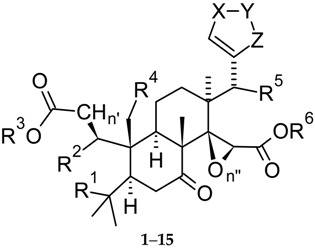
Overview of the main structural features of compounds **1**–**15**.

Compound	Name	Molecular Formula	n′	n′′	R^1^	R^2^	R^3^	R^4^	R^5^	R^6^	X	Y	Z	Main Functional Groups	Notes
**1**	Limonin	C_26_H_30_O_8_	2	1	O		Cycle bond		Cycle bond		O–CH=CH			Dilactone	-
**2**	Nomilin	C_28_H_34_O_9_	2	1	Cycle bond	OAc	Cycle bond	H	Cycle bond		O–CH=CH			Dilactone, acetic ester	-
**3**	Obacunone	C_26_H_30_O_7_	1	1	Cycle bond	-	Cycle bond	H	Cycle bond		O–CH=CH			Dilactone	Dehydrated analog of **4**
**4**	Deacetylnomilin	C_26_H_32_O_8_	2	1	Cycle bond	OH	Cycle bond	H	Cycle bond		O–CH=CH			Dilactone	Deacetyl-derivative of **2**
**5**	Limonexic acid	C_26_H_30_O_10_	2	1	O		Cycle bond		Cycle bond		CO–O–CHOH			Dilactone, pseudoacid	Hydroxybutenolide analog of **1**
**6**	Isolimonexic acid	C_26_H_30_O_10_	2	1	O		Cycle bond		Cycle bond		CHOH–O–CO			Dilactone, pseudoacid	Constitutional isomer of **5**
**7**	Citrusin	C_28_H_34_O_11_	2	1	Cycle bond	OAc	Cycle bond	H	Cycle bond		CHOH–O–CO			Dilactone, acetic ester, pseudoacid	Hydroxybutenolide analog of **2**
**8**	Ichangin	C_26_H_32_O_9_	2	1	OH	OH	Cycle bond		Cycle bond		O–CH=CH			Dilactone	Spiro analog of **1**
**9**	Isoobacunoic acid	C_26_H_32_O_8_	2	1	O		H	H	Cycle bond		O–CH=CH			Lactone, carboxylic acid	Product of formal reductive cleavage of **1**
**10**	Deoxylimonin	C_26_H_30_O_7_	2	0	O		Cycle bond		Cycle bond		O–CH=CH			Dilactone	Deoxidized analog of **1**
**11**	Limonin 17-β-d-glucoside	C_32_H_42_O_14_	2	1	O		Cycle bond		Oglc ^1^	H	O–CH=CH			Lactone, carboxylic acid	17-β-d-Glucopyranoside of **1**
**12**	Obacunone 17-β-d-glucoside	C_32_H_42_O_13_	1	1	Cycle bond	-	Cycle bond	H	Oglc ^1^	H	O–CH=CH			Lactone, carboxylic acid	17-β-d-Glucopyranoside of **3**
**13**	Deacetylnomilinic acid 17-β-d-glucoside	C_32_H_46_O_15_	2	1	OH	OH	H	H	Oglc ^1^	H	O–CH=CH			Dicarboxylic acid	17-β-d-Glucopyranoside of hydrolyzed **4**
**14**	Nomilinic acid 17-β-d-glucoside	C_34_H_48_O_16_	2	1	OH	OAc	H	H	Oglc ^1^	H	O–CH=CH			Dicarboxylic acid, acetic ester	Ac-derivative of **13**
**15**	Isoobacunoic acid 17-β-d-glucoside	C_32_H_44_O_14_	2	1	O		H	H	Oglc ^1^	H	O–CH=CH			Dicarboxylic acid	17-β-d-Glucopyranoside of **9**

^1^ Oglc: *O*-β-d-glucopyranosyl.

**Table 2:** Overview of the main physico-chemical properties and biological activities of compounds **1**–**15**.

Compound	Name (Other Trivial Names)	CAS Number	MW	Acidity ^1^	Log *P* ^1^	Log *D* ^1^ (pH 7.4)	Most Studied Biological Activities
**1**	Limonin (citrolimonin, dictamnolactone, evodin, obaculactone)	1180-71-8	470	p*K*_a_ > 8	1.66	1.66	Analgesic [45], anticancer [46,47,48,49,50,51,52,53,54,55,56,57,58,59], anti-inflammatory [45,60,61,62], antibacterial [63,64], antioxidant [4,65,66], antiviral [67], larvicidal [68,69]
**2**	Nomilin	1063-77-0	514	p*K*_a_ > 8	2.47	2.47	Anticancer [46,47,58,59,70], anti-hyperglycemic [71], anti-inflammatory [25,62], antioxidant [4,65,66], antiviral [67], larvicidal [68,69], osteoclastogenesis inhibition [72]
**3**	Obacunone (casimirolide, obacunon, tricoccin S_3_)	753-03-1	454	p*K*_a_ > 8	2.91	2.91	Anticancer [46,47,58,73,74,75], antibacterial [76,77] anti-hyperglycemic [78], antioxidant [66], larvicidal [79,80]
**4**	Deacetylnomilin (isolimonin, deacetylnomilinate)	3264-90-2	472	p*K*_a_ > 8	1.57	1.57	Anticancer [58,70], antioxidant [66]
**5**	Limonexic acid (limonexin, shihulimonin A, substance X) ^2^	99026-99-0	502	6 ≤ p*K*_a_ ≤ 8	−0.93	−0.7	Anticancer [28,48,52,81], antioxidant [28], hepatoprotection [82]
**6**	Isolimonexic acid ^3^	113164-90-2	502	6 ≤ p*K*_a_ ≤ 8	−0.93	−0.7	Anticancer [48,52]
**7**	Citrusin ^3^	108943-57-3	546	6 ≤ p*K*_a_ ≤ 8	−0.04	0.24	Anti-inflammatory [25]
**8**	Ichangin	10171-61-6	488	p*K*_a_ > 8	0.13	0.13	Antibacterial [83,84]
**9**	Isoobacunoic acid (iso-obacunoic acid)	751-28-0	472	4 ≤ p*K*_a_ < 6	2.33	−1.03	Antibacterial [83]
**10**	Deoxylimonin (desoxylimonin)	989-23-1	454	p*K*_a_ > 8	2.34	2.34	Analgesic [45], anti-inflammatory [45]
**11**	Limonin 17-β-d-glucoside (limonin 17-β-d-glucopyranoside, glucosyl-limonin)	123564-61-4	650	4 ≤ p*K*_a_ < 6	−0.46	−3.66	Anticancer [52,85,86,87], antioxidant [66,86], hepatoprotection [88]
**12**	Obacunone 17-β-d-glucoside (obacunone 17-β-d-glucopyranoside) ^4^	123564-64-7	635	4 ≤ p*K*_a_ < 6	0.79	−2.61	Anticancer [75,76,86,89], antioxidant [86,89]
**13**	Deacetylnomilinic acid 17-β-d-glucoside (deacetylnomilinic acid 17-β-d-glucopyranoside) ^4^	125107-16-6	671	p*K*_a_ < 4	−2.2	−5.9	Anticancer [89], antioxidant [89]
**14**	Nomilinic acid 17-β-d-glucoside (nomilinic acid 17-β-d-glucopyranoside) ^4^	125107-15-5	713	p*K*_a_ < 4	0.04	−4.42	Anticancer [46,89], antioxidant [89]
**15**	Isoobacunoic acid 17-β-d-glucoside (isoobacunoic acid 17-β-d-glucopyranoside) ^4^	125225-95-8	653	p*K*_a_ < 4	0.21	−3.49	Antibacterial [83]

^1^ Predicted data generated using the ACD/Labs Percepta Platform—PhysChem Module (ACD/Labs, Toronto, ON, Canada); ^2^ Experimental; ^3^ p*K*_a_ was assumed to be similar to that of limonexic acid (5); ^4^ pKa value from ChemAxon (https://chemicalize.com/#/calculation).

## References

[1] Manners G.D. (2007). Citrus limonoids: Analysis, bioactivity, and biomedical prospects. J. Agric. Food Chem..

[2] Wang S., Tu H., Wan J., Chen W., Liu X., Luo J., Xu J., Zhang H. (2016). Spatio-temporal distribution and natural variation of metabolites in Citrus fruits. Food Chem..

[3] Hamdan D., El-Readi M.Z., Tahrani A., Herrmann F., Kaufmann D., Farrag N., El-Shazly A., Wink M. (2011). Secondary metabolites of ponderosa lemon (*Citrus*
*pyriformis*) and their antioxidant, anti-inflammatory, and cytotoxic activities. Z. Naturforsch..

[4] Hamdan D., El-Readi M.Z., Tahrani A., Herrmann F., Kaufmann D., Farrag N., El-Shazly A., Wink M. (2011). Chemical composition and biological activity of *Citrus jambhiri* Lush. Food Chem..

[5] Russo M., Arigò A., Calabrò M.L., Farnetti S., Mondello L., Dugo P. (2016). Bergamot (*Citrus bergamia*
*Risso*) as a source of nutraceuticals: Limonoids and flavonoids. J. Funct. Food.

[6] Nakagawa H., Takaishi Y., Tanaka N., Tsuchiya K., Shibata H., Higuti T. (2006). Chemical constituents from the peels of *Citrus sudachi*. J. Nat. Prod..

[7] Yang Y., Zhao X.J., Pan Y., Zhou Z. (2016). Identification of the chemical compositions of Ponkan peel by ultra performance liquid chromatography coupled with quadrupole time-of-flight mass spectrometry. Anal. Methods.

[8] Hasegawa S., Berhow M.A., Hasegawa S., Manners G.D., ACS Symposium Series (2000). Biochemistry of Limonoids. Citrus Limonoids.

[9] Hasegawa S., Miyake M. (1996). Biochemistry and biological functions of Citrus limonoids. Food Rev. Int..

[10] Glabasnia A., Hofmann T. (2008). On the non-enzymatic liberation of limonin and C17-epilimonin from limonin-17-β-d-glucopyranoside in orange juice. Eur. Food Res. Technol..

[11] Li S., Wang Z., Ding F., Sun D., Ma Z., Cheng Y., Xu J. (2014). Content changes of bitter compounds in “Guoqing No. 1” Satsuma mandarin (*Citrus unshiu* Marc.) during fruit development of consecutive 3 seasons. Food Chem..

[12] Baldwin E., Plotto A., Manthey J., McCollum G., Bai J., Irey M., Cameron R., Luzio G. (2010). Effect of *Liberibacter* infection (Huanglongbing disease) of citrus on orange fruit physiology and fruit/fruit juice quality: Chemical and physical analyses. J. Agric. Food Chem..

[13] Raithore S., Dea S., McCollum G., Manthey J.A., Bai J., Leclair C., Hijaz F., Narciso J.A., Baldwin E.A., Plotto A. (2016). Development of delayed bitterness and effect of harvest date in stored juice from two complex citrus hybrids. J. Sci. Food Agric..

[14] Karim M.R., Hashinaga F. (2010). Possible role of carboxyl and imidazole groups in the catalysis of pummelo limonoid glucosyltransferase. Chin. J. Catal..

[15] Roy A., Saraf S. (2006). Limonoids: Overview of significant bioactive triterpenes distributed in plants kingdom. Biol. Pharm. Bull..

[16] Todaro A., Palmeri R., Scalone D., Alberio G.R.A., Serafini M., Spagna G. (2013). Removal of bitter compounds from citrus byproducts. Ital. J. Food Sci..

[17] Verma J.P., Singh S., Ghosh M., Srivastava P.K. (2010). Identification and characterization of cellular locus of limonin biotransforming enzyme in *Pseudomonas putida*. Int. J. Food Sci. Technol..

[18] Patil B.S., Yu J., Dandekar D.V., Toledo R.T., Singh R.K., Pike L.M., Patil B.S., Turner N.D., Miller E.G., Brodbelt J.S. (2006). Citrus bioactive limonoids and flavonoids extraction by supercritical fluids. Potential Health Benefits of Citrus.

[19] Chaudhary P.R., Jayaprakasha G.K., Patil B.S., Porat R. (2012). Grapefruit degreening influence on health promoting limonoids and flavoniods. Acta Hortic..

[20] Chaudhary P.R., Jayaprakasha G.K., Patil B.S. (2015). Ethylene degreening modulates health promoting phytochemicals in Rio Red grapefruit. Food Chem..

[21] Chaudhary P., Jayaprakasha G.K., Porat R., Patil B.S. (2012). Degreening and postharvest storage influences “Star Ruby” grapefruit (*Citrus*
*paradisi* Macf.) bioactive compounds. Food Chem..

[22] Bai J., Manthey J.A., Ford B.L., Luzio G., Cameron R.G., Narciso J., Baldwin E.A. (2013). Effect of extraction, pasteurization and cold storage on flavonoids and other secondary metabolites in fresh orange juice. J. Sci. Food Agric..

[23] Ram L., Dinesh K. (2012). Effect of growth stages on the changes in bioactive compounds of Nagpur mandarin (*Citrus reticulata*) fruits of *Ambia* crops. Indian J. Agric. Sci..

[24] Arias B.Á., Ramón-Laca L. (2005). Pharmacological properties of citrus and their ancient and medieval uses in the Mediterranean region. J. Ethnopharmacol..

[25] Chan Y.-Y., Li C.-H., Shen Y.-C., Wu T.-S. (2010). Anti-inflammatory principles from the stem and root barks of *Citrus medica*. Chem. Pharm. Bull..

[26] Xu H., Li Q., Yin Y., Lv C., Sun W., He B., Liu R., Chen X., Bi K. (2013). Simultaneous determination of three alkaloids, four ginsenosides and limonin in the plasma of normal and headache rats after oral administration of Wu-Zhu-Yu decoction by a novel ultra fast liquid chromatography-tandem mass spectrometry method: Application to a comparative pharmacokinetics and ethological study. J. Mass Spectrom..

[27] Lv M., Tian Y., Zhang Z., Liang J., Xu F., Sun J. (2015). Plant metabolomics driven chemical and biological comparison of the root bark of *Dictamnus dasycarpus* and *Dictamnus angustifolius*. RSC Adv..

[28] Zhao H.Y., Yang L., Wei J., Huang M., Jiang J.-G. (2012). Bioactivity evaluations of ingredients extracted from the flowers of *Citrus aurantium* L. var. *amara* Engl.. Food Chem..

[29] Patil B.S., Jayaprakasha G.K., Chidambara Murthy K.N., Vikram A. (2009). Bioactive compounds: Historical perspectives, opportunities, and challenges. J. Agric. Food Chem..

[30] Codoner-Franch P., Valls-Belles V. (2010). Citrus as functional foods. Curr. Top. Nutraceutical Res..

[31] Zou Z., Xi W., Hu Y., Nie C., Zhou Z. (2016). Antioxidant activity of citrus fruits. Food Chem..

[32] Kaur J., Kaur G. (2015). An insight into the role of citrus bioactives in modulation of colon cancer. J. Funct. Food.

[33] Harris E.D., Poulose S.M., Patil B. (2007). Citrus limonoids are unique andeffective anticancer agents. Acta Hortic..

[34] Sato R. (2013). Nomilin as an anti-obesity and anti-hyperglycemic agent. Vitam. Horm..

[35] Ejaz S., Ejaz A., Matsuda K., Lim C.W. (2006). Limonoids as cancer chemopreventive agents. J. Sci. Food Agric..

[36] Patil B.S., Brodbelt J.S., Miller E.G., Turner N.D., Patil B.S., Turner N.D., Miller E., Brodbelt J. (2006). Potential health benefits of citrus: An overview. Potential Health Benefits of Citrus.

[37] Heasley B. (2011). Synthesis of limonoid natural products. Eur. J. Org. Chem..

[38] Nikkon A., Silversmith E.F. (1987). Appendix A. Brief etymology of some traditional chemical names. The Name Game.

[39] Ruzicka L. (1953). The isoprene rule and the biogenesis of terpenic compounds. Experientia.

[40] Giles P.M. (1999). Revised Section F: Natural products and related compounds. Pure Appl. Chem..

[41] Behenna D.C., Corey E.J. (2008). Simple enantioselective approach to synthetic limonoids. J. Am. Chem. Soc..

[42] Okogun J.I., Fakunle C.O., Ekong D.E.U. (1975). Chemistry of the meliacins (limonoids). The structure of melianin A, a new protomeliacin from *Melia azedarach*. J. Chem. Soc. Perkin I.

[43] Lakshmi V., Gupta P. (2008). An overview of the genus *Xylocarpus*. Nat. Prod. Res..

[44] Fraser L.A., Mulholland D.A., Fraser D.D. (1997). Classification of limonoids and protolimonoids using neural networks. Phytochem. Anal..

[45] Yang Y., Wang X., Zhu Q., Gong G., Luo D., Jiang A., Yang L., Xu Y. (2014). Synthesis and pharmacological evaluation of novel limonin derivatives as anti-inflammatory and analgesic agents with high water solubility. Bioorg. Med. Chem. Lett..

[46] Kim J., Jayaprakasha G.K., Patil B.S. (2013). Limonoids and their anti-proliferative and anti-aromatase properties in human breast cancer cells. Food Funct..

[47] Poulose S.M., Jayaprakasha G.K., Mayer R.T., Girennavar B., Patil B.S. (2007). Purification of citrus limonoids and their differential inhibitory effects on human cytochrome P450 enzymes. J. Sci. Food Agric..

[48] Patil J.R., Chidambara Murthy K.N., Jayaprakasha G.K., Chetti M.B., Patil B.S. (2009). Bioactive compounds from mexican lime (*Citrus aurantifolia*) juice induce apoptosis in human pancreatic cells. J. Agric. Food Chem..

[49] Perez J.L., Jayaprakasha G.K., Valdivia V., Munoz D., Dandekar D.V., Ahmad H., Patil B.S. (2009). Limonin methoxylation influences the induction of glutathione *S*-transferase and quinone reductase. J. Agric. Food Chem..

[50] Vanamala J., Leonardi T., Patil B.S., Taddeo S.S., Murphy M.E., Pike L.M., Chapkin R.S., Lupton J.R., Turner N.D. (2006). Suppression of colon carcinogenesis by bioactive compounds in grapefruit. Carcinogenesis.

[51] El-Readi M.Z., Hamdan D., Farrag N., El-Shazly A., Wink M. (2010). Inhibition of P-glycoprotein activity by limonin and other secondary metabolites from *Citrus* species in human colon and leukaemia cell lines. Eur. J. Pharmacol..

[52] Patil J.R., Jayaprakasha G.K., Chidambara Murthy K.N., Chetti M.B., Patil B.S. (2010). Characterization of *Citrus aurantifolia* bioactive compounds and their inhibition of human pancreatic cancer cells through apoptosis. Microchem. J..

[53] Han Y.L., Yu H.L., Li D., Meng X.L., Zhou Z.Y., Yu Q., Zhang X.Y., Wang F.J., Guo C. (2011). Inhibitory effects of limonin on six human cytochrome P450 enzymes and P-glycoprotein in vitro. Toxicol. In Vitro.

[54] Shimizu S., Miyamoto S., Fujii G., Nakanishi R., Onuma W., Ozaki Y., Fujimoto K., Yano T., Mutoh M. (2015). Suppression of intestinal carcinogenesis in Apc-mutant mice by limonin. J. Clin. Biochem. Nutr..

[55] Langeswarana K., Kumar S.G., Perumal S., Revathy R., Balasubramaniam M.P. (2013). Limonin—A citrus limonoid, establish anticancer potential by stabilizing lipid peroxidation and antioxidant status against *N*-nitrosodiethylamine induced experimental hepatocellular carcinoma. Biomed. Prev. Nutr..

[56] Das A., Miller R., Lee P., Holden C.A., Lindhorst S.M., Jaboin J., Vandergrift W.A., Banik N.L., Giglio P., Varma A.K. (2015). A novel component from citrus, ginger, and mushroom family exhibits antitumor activity on human meningioma cells through suppressing the Wnt/β-catenin signaling pathway. Tumor Biol..

[57] Chidambara Murthy K.N., Jayaprakasha G.K., Patil B.S. (2013). Citrus limonoids and curcumin additively inhibit human colon cancer cells. Food Funct..

[58] Poulose S.M., Harris E.D., Patil B.S. (2006). Antiproliferative effects of citrus limonoids against human neuroblastoma and colonic adenocarcinoma cells. Nutr. Cancer.

[59] Ahmad H., Li J., Polson M., Mackie K., Quiroga W., Patil B.S. (2006). Citrus limonoids and flavonoids: Enhancement of phase II detoxification enzymes and their potential in chemoprevention. ACS Symp. Ser..

[60] Mahmoud M.F., Hamdan D.I., Wink M., El-Shazly A.M. (2014). Hepatoprotective effect of limonin, a natural limonoid from the seed of *Citrus aurantium* var. bigaradia, on d-galactosamine-induced liver injury in rats. Naunyn Schmiedebergs Arch. Pharmacol..

[61] Mahmoud M.F., Gamal S., El-Fayoumi H.M. (2014). Limonin attenuates hepatocellular injury following liver ischemia and reperfusion in rats via toll like receptor dependent pathway. Eur. J. Pharmacol..

[62] Kim J., Jayaprakasha G.K., Muthuchamy M., Patil B.S. (2011). Structure-function relationships of citrus limonoids on p38 MAP kinase activity in human aortic smooth muscle cells. Eur. J. Pharmacol..

[63] Vikram A., Jayaprakasha G.K., Jesudhasan P.R., Pillai S.D., Patil B.S. (2012). Limonin 7-methoxime interferes with *Escherichia coli* biofilm formation and attachment in type 1 pili and antigen 43 dependent manner. Food Control.

[64] Ribeiro A.B., Abdelnur P.V., Garcia C.F., Belini A., Severino V.G.P., da Silva M.F., Fernandes J.B., Vieira P.C., de Carvalho S.A., de Souza A.A. (2008). Chemical characterization of *Citrus sinensis* grafted on *C. limonia* and the effect of some isolated compounds on the growth of *Xylella fastidiosa*. J. Agric. Food Chem..

[65] Pichaiyongvongdee S., Haruenkit R. (2009). Investigation of limonoids, flavanones, total polyphenol content and antioxidant activity in seven Thai pummelo cultivars. Kasetsart J. (Nat. Sci.).

[66] Mandadi K.K., Jayaprakasha G.K., Bhat N.G., Patil B.S. (2007). Red Mexican grapefruit: A novel source for bioactive limonoids and their antioxidant activity. Z. Naturforsch. C.

[67] Balestrieri E., Pizzimenti F., Ferlazzo A., Giofrè S.V., Iannazzo D., Piperno A., Romeo R., Chiacchio M.A., Mastino A., Macchi B. (2011). Antiviral activity of seed extract from *Citrus bergamia* towards human retroviruses. Bioorg. Med. Chem..

[68] Bilal H., Akram W., Ali-Hassan S. (2012). Larvicidal activity of citrus limonoids against *Aedes albopictus* larvae. J. Arthropod Borne Dis..

[69] Hafeez F., Akram W., Shaalan E.A. (2011). Mosquito larvicidal activity of citrus limonoids against *Aedes albopictus*. Parasitol. Res..

[70] Perez J.L., Jayaprakasha G.K., Cadena A., Martinez E., Ahmad H., Patil B.S. (2010). In vivo induction of phase II detoxifying enzymes, glutathione transferase and quinone reductase by citrus triterpenoids. BMC Complement. Altern. Med..

[71] Ono E., Inoue J., Hashidume T., Shimizu M., Sato R. (2011). Anti-obesity and anti-hyperglycemic effects of the dietary citrus limonoid nomilin in mice fed a high-fat diet. Biochem. Biophys. Res. Commun..

[72] Kimira Y., Taniuchi Y., Nakatani S., Sekiguchi Y., Kim H.J., Shimizu J., Ebata M., Wada M., Matsumoto A., Mano H. (2015). Citrus limonoid nomilin inhibits osteoclastogenesis in vitro by suppression of NFATc1 and MAPK signaling pathways. Phytomedicine.

[73] Murthy K.N., Jayaprakasha G.K., Patil B.S. (2015). Cytotoxicity of obacunone and obacunone glucoside in human prostate cancer cells involves Akt-mediated programmed cell death. Toxicology.

[74] Kim J., Jayaprakasha G.K., Patil B.S. (2014). Obacunone exhibits anti-proliferative and anti-aromatase activity in vitro by inhibiting the p38 MAPK signaling pathway in MCF-7 human breast adenocarcinoma cells. Biochimie.

[75] Murthy K.N., Jayaprakasha G.K., Patil B.S. (2011). Obacunone and obacunone glucoside inhibit human colon cancer (SW480) cells by the induction of apoptosis *Food Chem*. Toxicol..

[76] Vikram A., Jayaprakasha G.K., Jesudhasan P.R., Pillai S.D., Patil B.S. (2012). Obacunone represses salmonella pathogenicity Islands 1 and 2 in an envz-dependent fashion. Appl. Environ. Microbiol..

[77] Vikram A., Jesudhasan P.R., Jayaprakasha G.K., Pillai B.S., Patil B.S. (2010). Grapefruit bioactive limonoids modulate *E. coli* O157:H7 TTSS and biofilm. Int. J. Food Microbiol..

[78] Horiba T., Katsukawa M., Mita M., Sato R. (2015). Dietary obacunone supplementation stimulates muscle hypertrophy, and suppresses hyperglycemia and obesity through the TGR5 and PPARγ pathway. Biochem. Biophys. Res. Commun..

[79] Yu X., Ding G., Zhi X., Xu H. (2015). Insight into reduction of obacunone, and their ester derivatives as insecticidal agents against *Mythimna separata* Walker. Bioorg. Med. Chem. Lett..

[80] Yu X., Shi D., Zhi X., Li Q., Yao X., Xu H. (2015). Synthesis and quantitative structure-activity relationship (QSAR) study of C7-oxime ester derivatives of obacunone as insecticidal agents. RSC Adv..

[81] Kim J., Jayaprakasha G.K., Vikram A., Patil B.S. (2012). Methyl nomilinate from citrus can modulate cell cycle regulators to induce cytotoxicity in human colon cancer (SW480) cells in vitro. Toxicol. In Vitro.

[82] Lu Q., Yang L., Zhao H.Y., Jiang J.G., Xu H.L. (2013). Protective effect of compounds from the flowers of *Citrus aurantium* L. var. amara Engl against carbon tetrachloride-induced hepatocyte injury. Food Chem. Toxicol..

[83] Vikram A., Jesudhasan P.R., Jayaprakasha G.K., Pillai S.D., Patil B.S. (2011). Citrus limonoids interfere with *Vibrio harveyi* cell–cell signalling and biofilm formation by modulating the response regulator LuxO. Microbiology.

[84] Vikram A., Jesudhasan P.R., Pillai S.D., Patil B.S. (2012). Isolimonic acid interferes with *Escherichia coli* O157:H7 biofilm and TTSS in QseBC and QseA dependent fashion. BMC Microbiol..

[85] Zunino S.J., Storms D.H., Freytag T.L., Adkins Y.C., Bonnel E.L., Woodhouse L.R., Breksa A.P., Manners G.D., Mackey B.E., Kelley D.S. (2016). Dietary supplementation with purified citrus limonin glucoside does not alter ex vivo functions of circulating T lymphocytes or monocytes in overweight/obese human adults. Nutr. Res..

[86] Kim J., Jayaprakasha G.K., Uckoo R.M., Patil B.S. (2012). Evaluation of chemopreventive and cytotoxic effect of lemon seed extracts on human breast cancer (MCF-7) cells. Food Chem. Toxicol..

[87] Jayaprakasha G.K., Jadegoud Y., Nagana G., Patil B.S. (2010). Bioactive compounds from sour orange inhibit colon cancer cell proliferation and induce cell cycle arrest. J. Agric. Food Chem..

[88] Kelley D.S., Adkins Y.C., Zunino S.J., Woodhouse L.R., Bonnel E.L., Breksa A.P., Manners G.D., Mackey B.E. (2015). Citrus limonin glucoside supplementation decreased biomarkers of liver disease and inflammation in overweight human adults. J. Funct. Foods.

[89] Poulose S.M., Harris E.D., Patil B.S. (2005). Citrus limonoids induce apoptosis in human neuroblastoma cells and have radical scavenging activity. J. Nutr..

[90] Lv M., Xu P., Tian Y., Liang J., Gao Y., Xu F., Zhang Z., Sun J. (2015). Medicinal uses, phytochemistry and pharmacology of the genus Dictamnus (Rutaceae). J. Ethnopharmacol..

[91] Okamura H., Yamauchi K., Miyawaki K., Iwagawa T., Nakatani M. (1997). Synthesis and biological activities of degraded limonoids, (±)-fraxinellonone and its related compounds. Tetrahedron Lett..

[92] Tundis R., Loizzo M.R., Menichini F. (2014). An oerview on chemical aspects and potential health benefits of limonoids and their derivatives. Crit. Rev. Food Sci. Nutr..

[93] Fang X., Di Y.T., Hao X.J. (2011). The advances in the limonoid chemistry of the *Meliaceae* family. Curr. Org. Chem..

[94] Connolly J.D., Hill R.A. (2005). Triterpenoids. Nat. Prod. Rep..

[95] Connolly J.D., Hill R.A. (2007). Triterpenoids. Nat. Prod. Rep..

[96] Connolly J.D., Hill R.A. (2008). Triterpenoids. Nat. Prod. Rep..

[97] Connolly J.D., Hill R.A. (2010). Triterpenoids. Nat. Prod. Rep..

[98] Hill R.A., Connolly J.D. (2011). Triterpenoids. Nat. Prod. Rep..

[99] Hill R.A., Connolly J.D. (2012). Triterpenoids. Nat. Prod. Rep..

[100] Hill R.A., Connolly J.D. (2013). Triterpenoids. Nat. Prod. Rep..

[101] Hill R.A., Connolly J.D. (2015). Triterpenoids. Nat. Prod. Rep..

[102] Hopkins A.L., Keserü G.M., Leeson P.D., Rees D.C., Reynolds C.H. (2014). The role of ligand efficiency metrics in drug discovery. Nat. Rev. Drug Discov..

[103] Shultz M.D. (2013). Setting expectations in molecular optimizations: Strengths and limitations of commonly used composite parameters. Bioorg. Med. Chem. Lett..

[104] Abad-Zapatero C., Metz J.T. (2005). Ligand efficiency indices as guideposts for drug discovery. Drug Discov. Today.

[105] Gualdani R., Cavalluzzi M.M., Lentini G. (2016). Recent trends in the discovery of small molecule blockers of sodium channels. Curr. Med. Chem..

[106] Ritchie T.J., Macdonald J.F. (2009). The impact of aromatic ring count on compound developability—Are too many aromatic rings a liability in drug design?. Drug Discov. Today.

[107] Ritchie T.J., Macdonald S.J.F., Young R.J., Pickett S.T. (2011). The impact of aromatic ring count on compound developability: Further insights by examining carbo- and hetero-aromatic and -aliphatic ring types. Drug Discov. Today.

[108] Lovering F., Bikker J., Humblet C. (2009). Escape from Flatland: Increasing saturation as an approach to improving clinical success. J. Med. Chem..

[109] Kingwell K. (2009). Medicinal chemistry: Exploring the third dimension. Nat. Rev. Drug Discov..

[110] Hann M.M. (2011). Molecular obesity, potency and other addictions in drug discovery. Med. Chem. Commun..

[111] Ikegami F. (2010). Phellodendri Cortex (Phellodendron Bark). Wakanyaku.

[112] Schechter M.S., Haller H.L. (1940). The identity of obaculactone, evodin and dictamnolactone with limonin. J. Am. Chem. Soc..

[113] Online Etymology Dictionary. http://www.etymonline.com/index.php?search=lemon&searchmode=none.

[114] Bernays A.J. (1841). Limonin. Justus Liebigs Ann. Chem..

[115] Hartog P.J., Lee S. (1901). Bernays, Albert James (DNB01). Dictionary of National Biography (1st Supplement) I.

[116] Phetkul U., Wanlaso N., Mahabusarakam W., Phongpaichit S., Carroll A.R. (2013). New acridone from the wood of *Citrus reticulata* Blanco. Nat. Prod. Res..

[117] Chan Y.Y., Wu T.S., Kuo Y.H. (2009). Chemical constituents and cytotoxicity from the stem bark of *Citrus medica*. Heterocycles.

[118] Panthong K., Srisud Y., Rukachaisirikul V., Hutadilok-Towatana N., Voravuthikunchai S.P., Tewtrakul S. (2013). Benzene, coumarin and quinolinone derivatives from roots of *Citrus hystrix*. Phytochemistry.

[119] Biavatti M.W., Vieira P.C., Silva M.D., Fernandes J.B., Albuquerque S. (2001). Limonoids from the endemic Brazilian species *Raulinoa echinata*. Z. Naturforsch..

[120] Chansriniyom C., Ruangrungsi N., Lipipun V., Kumamoto T., Ishikawa T. (2009). Isolation of acridone alkaloids and *N*-[(4-monoterpenyloxy)phenylethyl]-substituted sulfur-containing propanamide derivatives from *Glycosmis parva* and their anti-herpes simplex virus activity. Chem. Pharm. Bull..

[121] Feng T., Wang R.R., Cai X.H., Zheng Y.T., Luo X.D. (2010). Anti-human immunodeficiency virus-1 constituents of the bark of *Poncirus trifoliata*. Chem. Pharm. Bull..

[122] Sun J.B., Jiang N., Lv M.Y., Wang P., Xu F.G., Liang J.Y., Qu W. (2015). Limonoids from the root bark of *Dictamnus*
*angustifolius*: Potent neuroprotective agents with biometal chelation and halting copper redox cycling properties. RSC Adv..

[123] Lv M., Tang B., Teng J., Liang J., Xu F., Zhang Z., Sun J. (2015). Chemotaxonomic significance of limonoids and triterpenoids from *Dictamnus*
*angustifolius* G. Don ex Sweet. Biochem. Syst. Ecol..

[124] Emerson O.H. (1951). Bitter Principles of Citrus. II. Relation of Nomilin and Obacunone. J. Am. Chem. Soc..

[125] Min Y.D., Kwon H.C., Yang M.C., Lee K.H., Choi S.U., Lee K.R. (2007). Isolation of limonoids and alkaloids from Phellodendron amurense and their multidrug resistance (MDR) reversal activity. Arch. Pharm. Res..

[126] Jeong S.Y., Sang H.S., Young C.K. (2008). Neuroprotective limonoids of root bark of *Dictamnus dasycarpus*. J. Nat. Prod..

[127] Yang J.L., Liu L.L., Shi Y.P. (2011). Limonoids and quinoline alkaloids from Dictamnus dasycarpus. Planta Med..

[128] Choodej S., Sommit D., Pudhom K. (2013). Rearranged limonoids and chromones from *Harrisonia perforata* and their anti-inflammatory activity. Bioorg. Med. Chem. Lett..

[129] Garnier-Suillerot A. (1995). Impaired accumulation of drug in multidrug resistant cells. What are the respective contributions of the kinetics of uptake and of P-glycoprotein-mediated efflux of drug?. Curr. Pharm. Des..

[130] Koller G., Czerny H. (1936). Über das limonin, den bitterstoff der orangenkerne den bitterstoff der orangenkerne. Chem. Mon..

[131] Emerson O.H. (1948). The bitter principles of citrus fruit. I. Isolation of nomilin, a new bitter principle from the seeds of oranges and lemons. J. Am. Chem. Soc..

[132] Poulose S.M., Harris E.D., Patil B.S. (2006). Cytotoxic and antineoplastic effects of citrus limonoids against human neuroblastoma and colonic adenocarcinoma cells. FASEB J..

[133] Breksa A.P., Hidalgo M.B., Wong R.Y. (2008). Stability of limonin glucoside in beverage matrices. J. Sci. Food Agric..

[134] Ng K.M., Gray A.I., Waterman P.G., But P.P.H., Kong Y.C. (1987). Limonoids, alkaloids, and a coumarin from the root and stem barks of *Tetradium glabrifolium*. J. Nat. Prod..

[135] Gai L., Rao G., Song C., Hu Z. (2001). Studies on the chemical constituents of *Evodia rutaecarpa* (Juss.) Benth. var. officinalis (Dode) Huang. Acta Pharm. Sin..

[136] Melera A., Schaffner K., Arigoni D., Jeger O. (1957). Zur konstitution des limonins I. Über den verlauf der alkalischen hydrolyse von limonin und limonol. Helv. Chim. Acta.

[137] Dreyer D.L. (1965). Citrus bitter principles—II: Application of NMR to structural and stereochemical problems. Tetrahedron.

[138] Kondo Y., Suzuki H., Nozoe S. (1985). Two γ-hydroxybutenolides from the bark of *Phellodendron amurense* and photooxidation of limonoids. Yakugaku Zasshi J. Pharm. Soc. Jpn..

[139] Biavatti M.W., Westerlon R., Burger C., Mora T.C., De Souza M.M. (2007). Antinociceptive action of limonexic acid obtained from *Raulinoa echinata*. J. Pharm. Pharmacol..

[140] Biavatti M.W., Westerlon R., Vieira P.C., Silva M.F., Fernandes J.B., Penaflor M.F., Bueno O.C., Ellena J. (2005). Leaf-cutting ants toxicity of limonexic acid and degraded limonoids from *Raulinoa*
*echinata*. X-ray structure of epoxy-fraxinellone. J. Braz. Chem. Soc..

[141] Coy Barrera C.A., Coy Barrera E.D., Granados Falla D.S., Delgado Murcia G., Cuca Suarez L.E. (2011). Seco-limonoids and quinoline alkaloids from *Raputia heptaphylla* and their antileishmanial activity. Chem. Pharm. Bull..

[142] Breksa A.P., Dragull K., Wong R.Y. (2008). Isolation and identification of the first C-17 limonin epimer, epilimonin. J. Agric. Food Chem..

[143] Manners G., Jacob R.B., Breksa A.P., Schoch T.K., Hasegawa S. (2003). Bioavailability of citrus limonoids in humans. J. Agric. Food Chem..

[144] Jayaprakasha G.K., Mandadi K.K., Poulose S.M., Jadegoud Y., Nagana Gowda G.A., Patil B.S. (2008). Novel triterpenoid from *Citrus aurantium* L. possesses chemopreventive properties against human colon cancer cells. Bioorg. Med. Chem..

[145] Bennett R.D., Hasegawa S., Herman Z. (1989). Glucosides of acidic limonoids in Citrus. Phytochemistry.

[146] Dreyer D.L., Pickering M.V., Cohan P. (1972). Distribution of limonoids in the rutaceae. Phytochemistry.

[147] Herman Z., Fong C.H., Hasegawa S., Linskens H.F., Jackson J.F. (1992). Analysis of limonoids in citrus seeds. Modern Methods of Plant Analysis: Seed Analysis.

[148] Hasegawa S., Berhow M.A., Fong C.H., Linskens H.F., Jackson J.F. (1996). Analysis of bitter principles in *Citrus*. Modern Methods of Plant Analysis: Fruit Analysis.

[149] Jayaprakasha G.K., Brodbelt J.S., Bhat N.G., Patil B.S., Patil B.S., Turner N.D., Miller E., Brodbelt J. (2006). Methods for the separation of limonoids from Citrus. Potential Health Benefits of Citrus.

[150] Phetkul U., Phongpaichit S., Watanapokasin R., Mahabusarakam W. (2014). New depside from *Citrus reticulata* Blanco. Nat. Prod. Res..

[151] Abbasi S., Zandi P., Mirbagheri E. (2005). Quantitation of limonin in Iranian orange juice concentrates using high-performance liquid chromatography and spectrophotometric methods. Eur. Food Res. Technol..

[152] Zhao P., Duan L., Guo L., Dou L.L., Dong X., Zhou P., Li P., Liu E.H. (2015). Chemical and biological comparison of the fruit extracts of *Citrus wilsonii* Tanaka and *Citrus medica* L.. Food Chem..

[153] Chu J., Li S.L., Yin Z.Q., Ye W.C., Zhang Q.W. (2012). Simultaneous quantification of coumarins, flavonoids and limonoids in Fructus Citri Sarcodactylis by high performance liquid chromatography coupled with diode array detector. J. Pharm. Biomed. Anal..

[154] Bilal H., Akram W., Hassan S.A., Sahar S., Iqbal M.M. (2013). Determination of limonin and nomilin contents in different citrus cultivar using high performance liquid chromatography. Pak. J. Sci. Ind. Res. Ser. B Biol. Sci..

[155] Liu C., Yan F., Gao H., He M., Wang Z., Cheng Y., Deng X., Xu J. (2015). Features of citrus terpenoid production as revealed by carotenoid, limonoid and aroma profiles of two pummelos (*Citrus maxima*) with different flesh color. J. Sci. Food Agric..

[156] Sun C.D., Chen K.S., Chen Y., Chen Q.J. (2005). Contents and antioxidant capacity of limonin and nomilin in different tissues of citrus fruit of four cultivars during fruit growth and maturation. Food Chem..

[157] McIntosh C.A., Berhow M.A., Hasegawa S., Manners G.D. (2000). Quantification of limonin and limonoate A-ring monolactone during growth and development of citrus fruit and vegetative tissues by radioimmunoassay. Citrus Limonoids. Functional Chemicals in Agriculture and Foods.

[158] Hasegawa S., Ou P., Fong C.H., Herman Z., Coggins C.W., Atkin D.R. (1991). Changes in the limonoate A-ring lactone and limonin 17-β-d-glucopyranoside content of navel oranges during fruit growth and maturation. J. Agric. Food Chem..

[159] Fong C.H., Hasegawa S., Coggins C.W., Atkin D.R., Miyake M. (1992). Contents of limonoids and limonin 17-β-d-glucopyranoside in fruit tissue of Valencia orange during fruit growth and maturation. J. Agric. Food Chem..

[160] Breksa A.P., Zukas A.A., Manners G.D. (2005). Determination of limonoate and nomilinoate A-ring lactones in citrus juices by liquid chromatography–electrospray ionization mass spectrometry. J. Chromatogr. A.

[161] Raman G., Cho M., Brodbelt J.S., Patil B.S. (2005). Isolation and purification of closely related *Citrus* limonoid glucosides by flash chromatography. Phytochem. Anal..

[162] Breksa A.P., Hidalgo M.B., Yuen M.L. (2009). Liquid chromatography–electrospray ionisation mass spectrometry method for the rapid identification of citrus limonoid glucosides in citrus juices and extracts. Food Chem..

[163] Breksa A.P., Dragull K. (2009). Development and validation of a decigram-scale method for the separation of limonin from limonin glucoside by C18 flash chromatography. Food Chem..

[164] Vikram A., Jayaprakasha G.K., Patil B.S. (2007). Simultaneous determination of citrus limonoid aglycones and glucosides by high performance liquid chromatography. Anal. Chim. Acta.

[165] Jayaprakasha G.K., Dandekar D.V., Tichy S.E., Patil B.S. (2011). Simultaneous separation and identification of limonoids from citrus using liquid chromatography-collision-induced dissociation mass spectra. J. Sep. Sci..

[166] Breksa A.P., Ibarra P. (2007). Colorimetric method for the estimation of total limonoid aglycones and glucoside contents in citrus juices. J. Agric. Food Chem..

[167] Breksa A.P., Kahn T., Zukas A.A., Hidalgo M.B., Lee Yuen M. (2011). Limonoid content of sour orange varieties. J. Sci. Food Agric..

[168] Minamisawa M., Yoshida S., Uzawa A. (2014). The functional evaluation of waste yuzu (*Citrus junos*) seeds. Food Funct..

[169] Kuroyanagi M., Ishii H., Kawahara N., Sugimoto H., Yamada H., Okihara K., Shirota O. (2008). Flavonoid glycosides and limonoids from Citrus molasses. J. Nat. Med..

[170] Arbona V., Iglesias D.J., Gómez-Cadenas A. (2015). Non-targeted metabolite profiling of citrus juices as a tool for variety discrimination and metabolite flow analysis. BMC Plant Biol..

[171] Vaclavik L., Schreiber A., Lacina O., Cajka T., Hajslova J. (2012). Liquid chromatography–mass spectrometry-based metabolomics for authenticity assessment of fruit juices. Metabolomics.

[172] Pan Z., Li Y., Deng X., Xiao S. (2014). Non-targeted metabolomic analysis of orange (*Citrus sinensis* [L.] Osbeck) wild type and bud mutant fruits by direct analysis in real-time and HPLC-electrospray mass spectrometry. Metabolomics.

[173] Ledesma-Escobar C.A., Priego-Capote F., Luque de Castro M.D. (2015). Characterization of lemon (*Citrus limon*) polar extract by liquid chromatography–tandem mass spectrometry in high resolution mode. J. Mass Spectrom..

[174] Ren W., Li Y., Zuo R., Wang H.J., Si N., Zhao H.Y., Han L.Y., Yang J., Bian B.L. (2014). Species-related difference between limonin and obacunone among five liver microsomes and zebrafish using ultra-high-performance liquid chromatography coupled with a LTQ-Orbitrap mass spectrometer. Rapid Commun. Mass Spectrom..

[175] Ren W., Xin S.K., Han L.Y., Zuo R., Li Y., Gong M.X., Wei X.L., Zhou Y.Y., He J., Wang H.J. (2015). Comparative metabolism of four limonoids in human liver microsomes using ultra-high-performance liquid chromatography coupled with high-resolution LTQ-Orbitrap mass spectrometry. Rapid Commun. Mass Spectrom..

[176] Tian Q., Miller E.G., Jayaprakasha G.K., Patil B.S. (2007). An improved HPLC method for the analysis of citrus limonoids in culture media. J. Chromatogr. B.

[177] Habtemariam S. (2008). Activity-guided isolation and identification of antioxidant components from ethanolic extract of *Peltiphyllum peltatum* (Torr.) Engl.. Nat. Prod. Commun..

[178] Habtemariam S. (2008). Activity-guided isolation and identification of free Radical-scavenging components from ethanolic extract of Boneset (Leaves of *Eupatorium perfoliatum*). Nat. Prod. Commun..

[179] Habtemariam S. (2011). Methyl-3-*O*-Methyl Gallate and Gallic Acid from the Leaves of *Peltiphyllum peltatum:* Isolation and Comparative Antioxidant, Prooxidant, and Cytotoxic Effects in Neuronal Cells. J. Med. Food.

[180] Habtemariam S., Cowley R.A. (2012). Antioxidant and anti-α-glucosidase compounds from the rhizome of *Peltiphyllum peltatum* (Torr.) Engl.. Phytother. Res..

[181] Habtemariam S., Dagne E. (2010). Comparative antioxidant, prooxidant and cytotoxic activity of sigmoidin A and eriodictyol. Planta Med..

[182] Habtemariam S. (2015). Investigation into the antioxidant and antidiabetic potential of *Moringa stenopetala*: Identification of the active principles. Nat. Prod. Commun..

[183] Habtemariam S., Varghese G.K. (2015). A Novel Diterpene Skeleton: Identification of a highly aromatic, cytotoxic and antioxidant 5-methyl-10-demethyl-abietane-type diterpene from *Premna serratifolia*. Phytother. Res..

[184] Juan-Badaturugea M., Habtemariam S., Jackson C., Thomas M.J.K. (2009). Antioxidant principles of *Tanacetum vulgare* L. aerial part. Nat. Prod. Commun..

[185] Juan-Badaturuge M., Habtemariam S., Thomas M.J.K. (2011). Antioxidant compounds from a South Asian beverage and medicinal plant, *Cassia auriculata*. Food Chem..

[186] Roselli M., Lentini G., Habtemariam S. (2012). Phytochemical, antioxidant and anti-alpha-glucosidase activity evaluations of *Bergenia cordifolia*. Phytother. Res..

[187] Habtemariam S., Dagne E. (2009). Differential cytotoxic and prooxidnant activity of knipholone and knipholone anthrone. Planta Med..

[188] Habtemariam S. (1997). Cytotoxicity and immunosuppressive activity of withanolides from *Discopodium penninervium*. Planta Med..

[189] Habtemariam S. (1995). Cytotoxicity of diterpenes from *Premna schimperi* and *Premna oligotricha*. Planta Med..

[190] Habtemariam S. (2003). Cytotoxic and cytostatic activity of erlangerins from *Commiphora erlangeriana*. Toxicon.

[191] Mireku E.A., Mensah A.Y., Mensah M.L.K., Tocher D.A., Habtemariam S. (2014). Anti-inflammatory properties of the stem-bark of *Anopyxis kaline*ana and its major constituent, methyl angolensate. Phytother. Res..

[192] Cavalluzzi M.M., Viale M., Bruno C., Carocci A., Catalano A., Carrieri A., Franchini C., Lentini G. (2013). A convenient synthesis of lubeluzole and its enantiomer: Evaluation as chemosensitizing agents on human ovarian adenocarcinoma and lung carcinoma cells. Bioorg. Med. Chem. Lett..

[193] Yamashita S., Naruko A., Nakazawa Y., Zhao L., Hayashi Y., Hirama M. (2015). Total synthesis of limonin. Angew. Chem. Int. Ed..

[194] Xiang Y., Cao J., Luo F., Wang D., Chen W., Li X., Sun C., Chen K. (2014). Simultaneous purification of limonin, nomilin and isoobacunoic acid from Pomelo Fruit (*Citrus grandis*) segment membrane. J. Food Sci..

[195] Rodríguez R.L.D., Jiménez R.A.A., Rueda L.E.A., Méndez A.J.J., Murillo A.W. (2014). Relationship between content of limonin in citrus waste and antifeedant activity against *Spodoptera frugiperda*. Rev. Colomb. Entomol..

